# SOX9 Governs Differentiation Stage-Specific Gene Expression in Growth Plate Chondrocytes via Direct Concomitant Transactivation and Repression

**DOI:** 10.1371/journal.pgen.1002356

**Published:** 2011-11-03

**Authors:** Victor Y. L. Leung, Bo Gao, Keith K. H. Leung, Ian G. Melhado, Sarah L. Wynn, Tiffany Y. K. Au, Nelson W. F. Dung, James Y. B. Lau, Angel C. Y. Mak, Danny Chan, Kathryn S. E. Cheah

**Affiliations:** 1 Department of Biochemistry, Li Ka Shing Faculty of Medicine, The University of Hong Kong, Hong Kong, China; 2 Centre for Reproduction, Development, and Growth, Li Ka Shing Faculty of Medicine, The University of Hong Kong, Hong Kong, China; Medical Research Council Human Genetics Unit, United Kingdom

## Abstract

Cartilage and endochondral bone development require SOX9 activity to regulate chondrogenesis, chondrocyte proliferation, and transition to a non-mitotic hypertrophic state. The restricted and reciprocal expression of the collagen X gene, *Col10a1*, in hypertrophic chondrocytes and *Sox9* in immature chondrocytes epitomise the precise spatiotemporal control of gene expression as chondrocytes progress through phases of differentiation, but how this is achieved is not clear. Here, we have identified a regulatory element upstream of *Col10a1* that enhances its expression in hypertrophic chondrocytes *in vivo*. In immature chondrocytes, where *Col10a1* is not expressed, SOX9 interacts with a conserved sequence within this element that is analogous to that within the intronic enhancer of the collagen II gene *Col2a1*, the known transactivation target of SOX9. By analysing a series of *Col10a1* reporter genes in transgenic mice, we show that the SOX9 binding consensus in this element is required to repress expression of the transgene in non-hypertrophic chondrocytes. Forced ectopic *Sox9* expression in hypertrophic chondrocytes *in vitro* and in mice resulted in down-regulation of *Col10a1*. Mutation of a binding consensus motif for GLI transcription factors, which are the effectors of Indian hedgehog signaling, close to the SOX9 site in the *Col10a1* regulatory element, also derepressed transgene expression in non-hypertrophic chondrocytes. GLI2 and GLI3 bound to the *Col10a1* regulatory element but not to the enhancer of *Col2a1*. In addition to *Col10a1*, paired SOX9–GLI binding motifs are present in the conserved non-coding regions of several genes that are preferentially expressed in hypertrophic chondrocytes and the occurrence of pairing is unlikely to be by chance. We propose a regulatory paradigm whereby direct concomitant positive and negative transcriptional control by SOX9 ensures differentiation phase-specific gene expression in chondrocytes. Discrimination between these opposing modes of transcriptional control by SOX9 may be mediated by cooperation with different partners such as GLI factors.

## Author Summary

Chondrogenic differentiation is a key process in the formation of endochondral bone. Despite the wealth of information about gene expression patterns and signaling pathways important for this process, it is not clear how differentiation state-specificity of transcription is controlled. The transcription factor SOX9 regulates chondrocyte differentiation, proliferation, and entry into hypertrophy and is highly expressed in immature/proliferating chondrocytes. It directly transactivates *Col2a1*, enhancing this gene's expression in immature/proliferating chondrocytes. The *Col10a1* gene is specifically expressed in hypertrophic chondrocytes in which *Sox9* is downregulated. How is differentiation phase-specific transcription of genes controlled in chondrocytes, particularly during hypertrophy? We found that SOX9 directly represses *Col10a1* expression in immature/proliferating chondrocytes of the growth plate, so that its expression is restricted to hypertrophic chondrocytes. Discrimination of this concomitant opposing transcriptional control may involve cooperation between SOX9 and different partners such as GLI factors (effectors of hedgehog signaling). SOX9 control of chondrocyte maturation therefore may be integrated with hedgehog signaling. Mutations in human *SOX9* cause the skeletal malformation syndrome campomelic dysplasia, which is attributed to the disruption of the chondrogenic differentiation program because of failure to express SOX9 target genes. This interpretation should be revised to include inappropriate expression of genes normally repressed by SOX9.

## Introduction

Chondrogenesis and the formation of bone by endochondral ossification depend on progressive steps of cell differentiation. Mesenchymal cells condense and differentiate into chondrocytes in a pattern that will define the eventual shape of the different skeletal elements. These chondrocytes proliferate, mature, exit the cell cycle and become prehypertrophic. The differentiation program culminates in the terminal differentiation and apoptosis of post-mitotic hypertrophic chondrocytes [1]. This differentiation program is controlled by members of the SOX and RUNX families of transcription factors and the integration of multiple signaling pathways mediated by Indian hedgehog (Ihh), parathyroid hormone-related protein (PTHrP), Wnts, BMPs, and Notch (reviewed in [2]). PTHrP and Ihh are two important players which interact to form a feedback loop that controls the pace of the differentiation program [3].

*Sox9* is essential for chondrogenesis and chondrocyte differentiation [4]–[6]. It is essential for mesenchymal condensation prior to chondrogenesis, and in its absence chondrocyte differentiation fails. Inactivation of *Sox9* in chondrocytes at different stages of differentiation suggests that its expression is essential for the survival of chondrocytes so that they can progress to hypertrophy [5]–[7]. Mutations in *SOX9* are associated with the human skeletal malformation syndrome, campomelic dysplasia, in which skeletal abnormalities can be attributed to the disruption of the chondrogenic differentiation program due to failure to express SOX9 target genes. Upon hypertrophy, chondrocytes down-regulate *Sox9* expression [8], [9], which is believed to mark the end of SOX9 control in the growth plate.

Despite the wealth of information about spatial and temporal gene expression patterns in the developing growth plate, it is not clear how transcriptional controls achieve appropriate and specific gene expression during chondrocyte differentiation. SOX9 activates many genes expressed in proliferating chondrocytes, including the extracellular matrix (ECM) genes *Col2a1*, *Col9a1*, *Col11a2*, *Acan* (aggrecan) and *Cd-rap*/*Mia1* [10]–[15]. For the *Col2a1* gene, which is expressed most strongly in proliferating chondrocytes, SOX9 directly transactivates the gene *in vivo* via a conserved enhancer sequence within the first intron [10], [11].

The collagen X gene, *Col10a1*, is a hypertrophic chondrocyte specific marker. The specificity and reciprocity of *Sox9* and *Col10a1* expression epitomise the strict control of temporal and differentiation phase-specific gene expression in the growth plate. *Col10a1* is ideal for studying transcriptional regulation because as well as its highly specific expression pattern, over-expression or loss-of-function does not disrupt chondrocyte differentiation. These properties simplify interpretation of changes in gene expression resulting from perturbing transcriptional control [16]–[18]. Here, we examined the transcriptional controls that restrict *Col10a1* expression to hypertrophic chondrocytes. We found that SOX9 coordinates gene expression during chondrocyte differentiation through both transcriptional activation and repression. Discrimination between these opposing actions is probably achieved by cooperation between SOX9 and different partners such as GLI factors.

## Results

### Proliferating and hypertrophic chondrocytes show overlapping and different protein binding domains in the *Col10a1* enhancer

**Figure 1:**
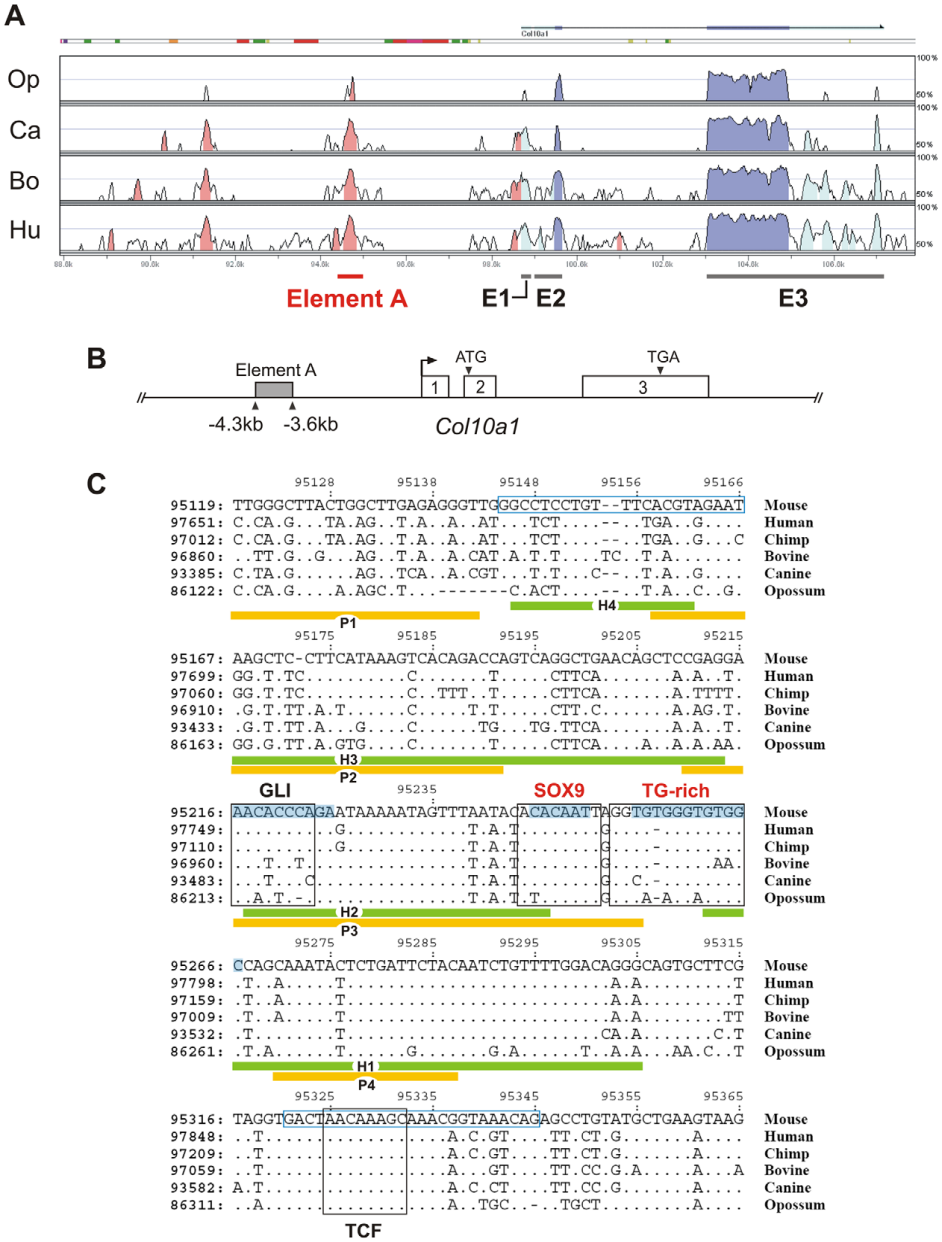
Conserved non-coding regions in *COL10A1*. (*A*) Global alignment of *COL10A1* loci was performed using mammalian genomes. The translated regions in exons 2 and 3 of *COL10A1* are highly conserved (deep blue). Element A of mouse *Col10a1*, which corresponds to the human enhancer, is located in the nearest conserved upstream non-coding region (pink). The untranslated regions (light blue) are poorly conserved in opossum. The mouse genome serves as the base sequence for alignment. Op: opossum; Ca: canine; Bo: bovine; Hu: human. (*B*) The 640 bp element A is located between −4286 and −3646 bp of mouse *Col10a1*. (*C*) Conserved non-coding regions in mammalian *COL10A1*. Alignment showed that the functional SOX9 binding site (COL2C1) is conserved in the mammalian *COL10A1* enhancers together with the adjacent TG-rich sequence. The positions of the chondrocyte-specific binding motifs H1–H4 and P1–P4 deduced from DNase I footprinting are indicated. The primer sequences used in the chromatin immunoprecipitation are indicated in blue boxes. Consensus GLI and TCF binding sites were identified near the SOX9/TG-rich motif. Sequences involved in mutagenesis are shaded in blue. Only the mismatched nucleotides are shown in the aligned sequence.

**Figure 2:**
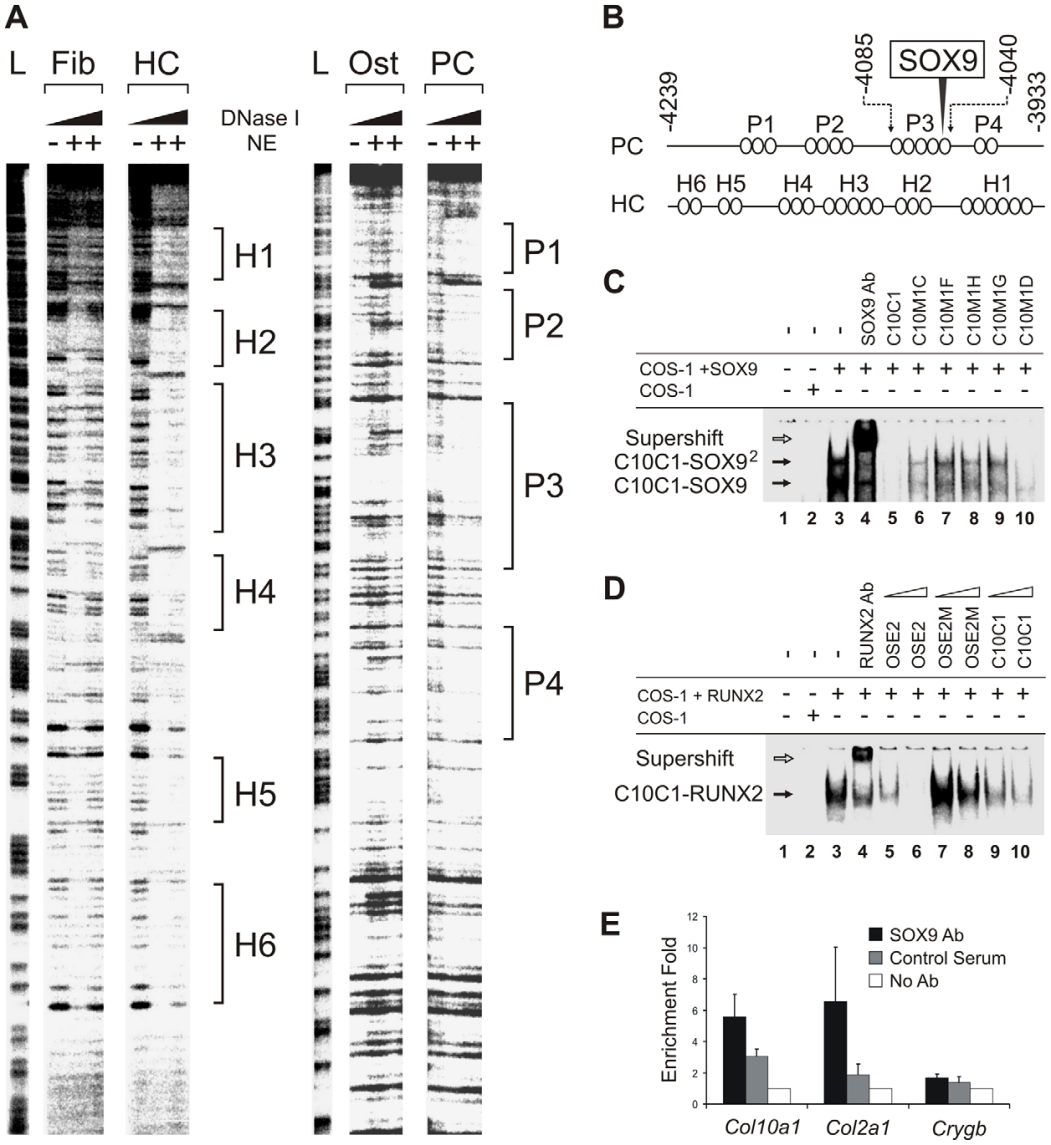
Interaction of SOX9 with a conserved upstream element of *Col10a1*. (*A*) A fragment of element A (−4239 to −3933 bp) was digested with increasing concentrations of DNase I in the presence of extract buffer alone or nuclear extracts (NE) from COS-1 (Fib), MCTs (HC), MC3T3-E1 (Ost), or CCL cells (PC), or used to produce the G/A ladder (L). (*B*) Diagram of interaction between cell type-specific nuclear factors (ovals) and element A based on the footprints. A SOX9 binding site is located in P3. (*C*) Electromobility shift assay (EMSA) of the interaction of the SOX9/TG-rich motif in element A of *Col10a1* (C10C1) with SOX9. The interaction involved dimeric SOX9 binding (C10C1-SOX9^2^). Complexes formed between SOX9 and C10C1 were challenged with SOX9 antibody (lane 4) and competitors of wild-type or mutant sequence (lane 5–10). Mutation of the SOX9 site and the adjacent TG-rich motif reduced SOX9 binding. (*D*) In EMSA, RUNX2 interacted with the *Bglap* (Osteocalcin) enhancer probe (OSE2) containing the osteoblast-specific RUNX2 binding consensus motif. The interaction was challenged with RUNX2 antibody (lane 4) and competitors of wild-type or mutant OSE2 (lane 5–8), or C10C1 (lane 9–10), at 10- or 100-fold excess. (*E*) Chromatin immunoprecipitation (ChIP) assay demonstrating SOX9 interaction with the SOX9/TG-rich binding site. Real-time PCR was performed to amplify element A of *Col10a1* as well as sequences from the *Col2a1* enhancer and *Crygb* (γ-b-crystallin) promoter. See also Figure S1.

Previous cell transfection studies identified an enhancer element upstream of human *COL10A1* [19]. This element is highly conserved in mammals and corresponds to a 640 bp region between −4.3 and −3.6 kb of the mouse *Col10a1* gene (designated element A) (Figure 1*A and 1B*). We used DNase I footprinting assays to test the configurations in which the element A sequences could be directly bound by nuclear factors derived from chondrocytes at different differentiation states (Figure 2*A*). Extracts from hypertrophic chondrocytes MCTs, but not fibroblasts COS-1 or osteoblasts MC3T3-E1, protected six blocks of sequence (H1–H6). Noticeably, four different blocks (P1–P4) that partially overlap H1–H4 were protected by extracts from the proliferating chondrocyte/chondrosarcoma cell line CCL (Figure 1*C* and Figure 2*A*). Since proliferating chondrocyte/chondrosarcoma cells do not express *Col10a1* (Figure S1 *A*), these results suggest that in these cells, the proteins that bind to element A may contribute to the repression of *Col10a1*.

### SOX9 binds to *Col10a1* element A in proliferating chondrocytes

We and others previously showed that SOX9 regulates *COL2A1*/*Col2a1* gene via a functional *in vivo* binding site in the intron 1 enhancer element [10], [11]. We identified the same SOX9-binding sequence within the *Col10a1* element A (Figure 1*C* and Figure 2*B*). This site, COL2C1, lies on a region in block P3 that is not protected in hypertrophic chondrocytes, and is adjacent to a stretch of thymidine/guanine-rich (TG-rich) sequence. Electromobility shift assays revealed that SOX9 bound to this SOX9/TG-rich motif with a similar affinity as to the *COL2A1* enhancer element (Figure S1 *C*, S1*E*, S1*F*) [10] and the interaction involved dimeric binding (Figure 2*C*, cf. lane 3–4). SOX9 also interacted with the TG-rich motif but with a lower affinity than with the consensus SOX9 site. Mutation of the TG-rich motif reduced the overall SOX9 binding to the P3 element (Figure 2*C*, cf. lanes 6–10). The TG-rich motif resembles a RUNX binding consensus sequence, but we found that RUNX2 did not interact with this motif effectively compared with its binding to the RUNX site in the *Bglap* (osteocalcin) enhancer (Figure 2*D*). Chromatin immunoprecipitation (ChIP) assays using extracts from E13.5 mouse limb, a stage at which the cartilage anlagen is largely composed of immature chondrocytes, confirmed specific SOX9 binding to the *Col10a1* element A and the *Col2a1* enhancer *in vivo* (Figure 2*E*).

### SOX9/TG-rich motif is required for appropriate *Col10a1* expression

**Figure 3:**
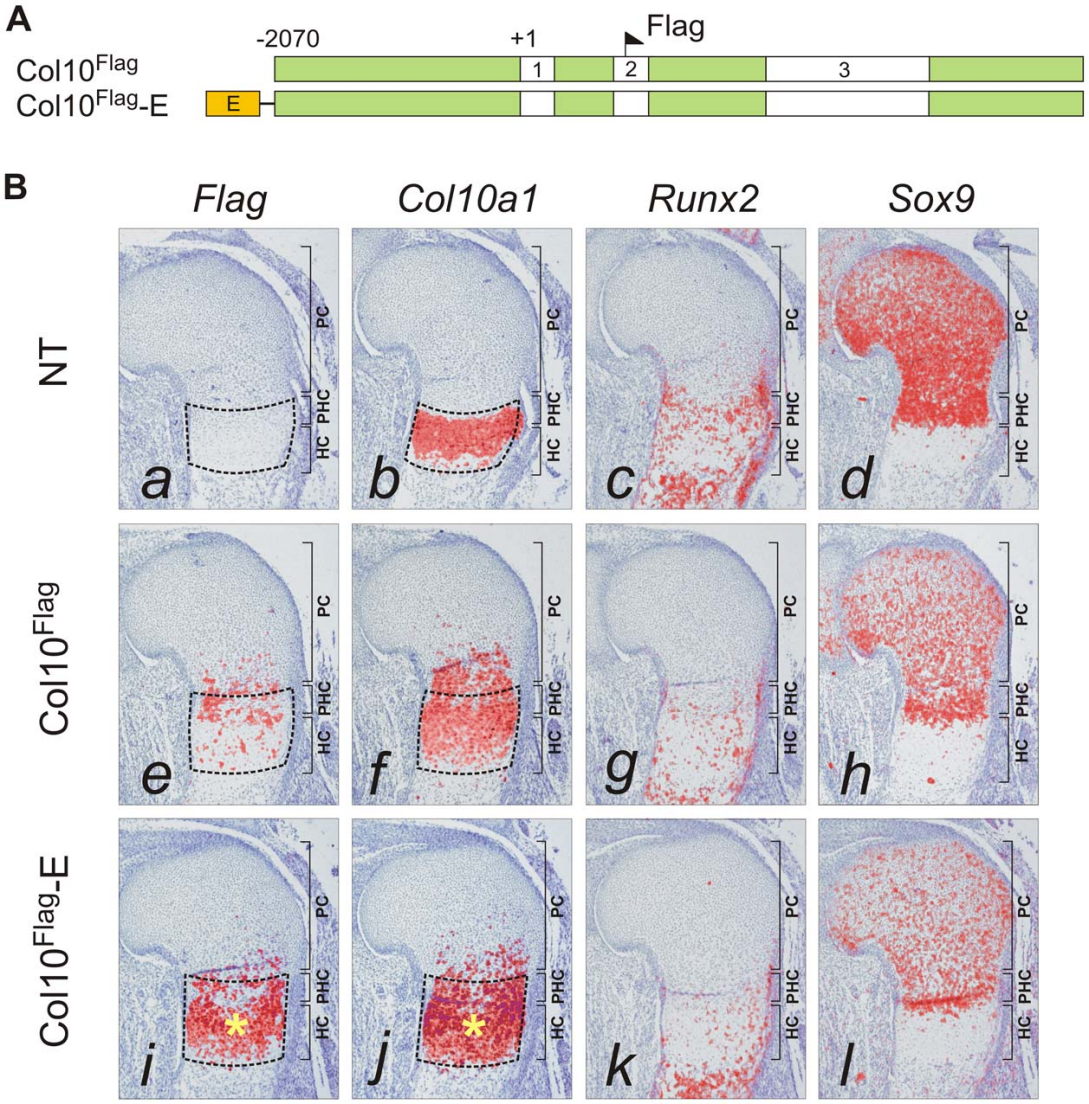
Element A enhances *Col10a1* transgene expression in the growth plate. (*A*) Diagram showing the Flag-tagged *Col10a1* mini-genes for transgenesis. The constructs included all introns and a 5′ flanking region up to −2.07 kb (green). At the 5′ end, the constructs were combined with the 640 bp element A (orange) containing wild-type sequence (E). (*B*) The expression pattern of the Flag-tagged *Col10a1* mini-genes (*a,e,i*) was compared with that of endogenous *Col10a1* (*b,f,j*), *Runx2* (*c,g,k*), and *Sox9* (*d,h,l*) by *in-situ* hybridization of proximal humerus in E15.5 expressing fetuses. (*a–d*) Tissue-specific *Col10a1* expression in hypertrophic zone of non-transgenic littermate (NT) was shown. (*e–h*) In the absence of element A, patchy expression of the mini-gene was observed in prehypertrophic and upper hypertrophic chondrocytes. (*i–l*) Addition of element A resulted in marked up-regulation of mini-gene expression in the whole hypertrophic zone (*). Since expression of *Col10a1* was used as readout of derepression, *Runx2* was used as an alternative marker of hypertrophic chondrocytes. In addition to hypertrophic chondrocytes, expression of *Runx2* was noted in the trabecular bone and in the prehypertrophic chondrocytes. The prehypertrophic and hypertrophic zones (encircled) were identified based on *Runx2* expression. PC, proliferating chondrocytes; PHC, prehypertrophic chondrocytes; HC, hypertrophic chondrocytes.

**Figure 4:**
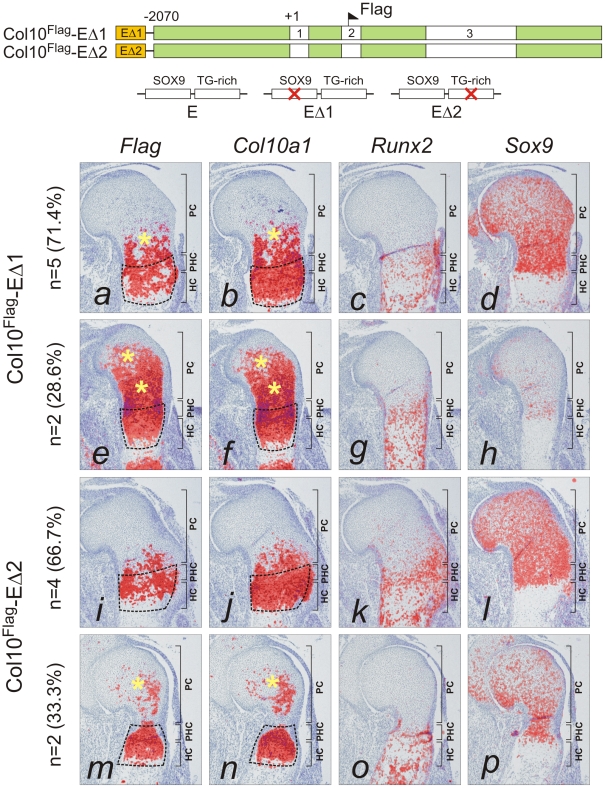
Mutation of SOX9 binding consensus results in derepression of *Col10a1* transgene expression *in vivo*. The expression pattern of mutant Flag-tagged *Col10a1* mini-genes, in which the SOX9 site (Col10^Flag^-EΔ1) or the TG-rich motif (Col10^Flag^-EΔ2) within the element A was mutated, was examined by *in-situ* hybridization of proximal humeri in E15.5 expressing fetuses. In a major (5/7) portion of Col10^Flag^-EΔ1 fetuses (*a–d*), the expression in the proliferating zone (*) was relatively higher than in the hypertrophic zone. In a minor (2/7) portion (*e–h*), the expression was predominant in nearly all immature chondrocytes (*). For Col10^Flag^-EΔ2, a minor (2/6) portion of fetuses (*m–p*) showed up-regulated expression in the non-hypertrophic zone (*), while a major (4/6) portion showed relatively slight mis-expression (*i–l*). The prehypertrophic and hypertrophic zones are encircled. PC, proliferating chondrocytes; PHC, prehypertrophic chondrocytes; HC, hypertrophic chondrocytes.

The paired SOX9 binding sequences in element A are separated by 4 bp, a distance similar to that between the paired SOX-like consensus sequences in *Col2a1*, *Col9a1*, and *Acan* that mediate transactivation of expression [15]. We tested the *in vivo* role of element A and the effects of SOX9/TG-rich motif mutations on the expression of *Col10a1* mini-genes (Figure 3*A*) in transgenic mice. We have previously shown that a Flag-tagged *Col10a1* vector Col10^Flag^ (formerly known as FColX) is expressed in P10 hypertrophic chondrocytes [18]. Here, we show that in E15.5 humeri, the Col10^Flag^ transgene was expressed in islands in prehypertrophic and hypertrophic chondrocytes in the upper hypertrophic zone (Figure 3*B*, *e*). In two independent mouse lines, a transgene comprising element A fused to the Col10^Flag^ (Col10^Flag^-E) was expressed in a similar pattern as Col10^Flag^, but was significantly more strongly expressed than Col10^Flag^ in all hypertrophic chondrocytes (Figure 3*B*, *i*), reflecting the enhancer activity of element A (see also Figure S2). However, mutation of the SOX9 site in element A (Col10^Flag^-EΔ1) resulted in marked expansion of the expression domain of the transgene, extending from the hypertrophic zone to the proliferating zone in the majority of transgenic fetuses (71.4%) (compare Figure 4, *a* with Figure 3*B*, *i*) and in almost all the *Sox9*-expressing chondrocytes in the rest (Figure 4, *e*). Expansion of transgene expression in proliferating chondrocytes was also noted but was less marked when the TG-rich motif in element A was mutated (Col10^Flag^-EΔ2) (compare Figure 4, *m* and *i* with *a* and *e*). Mutation of either SOX9 or the TG-rich motif did not abrogate transgene expression in hypertrophic chondrocytes. Together, these observations suggest that element A contains both positive and negative regulatory sequences, and that mutations in the SOX9/TG-rich motif in element A might disrupt SOX9-mediated repression in immature chondrocytes.

### SOX9 is a negative regulator of *Col10a1*

**Figure 5:**
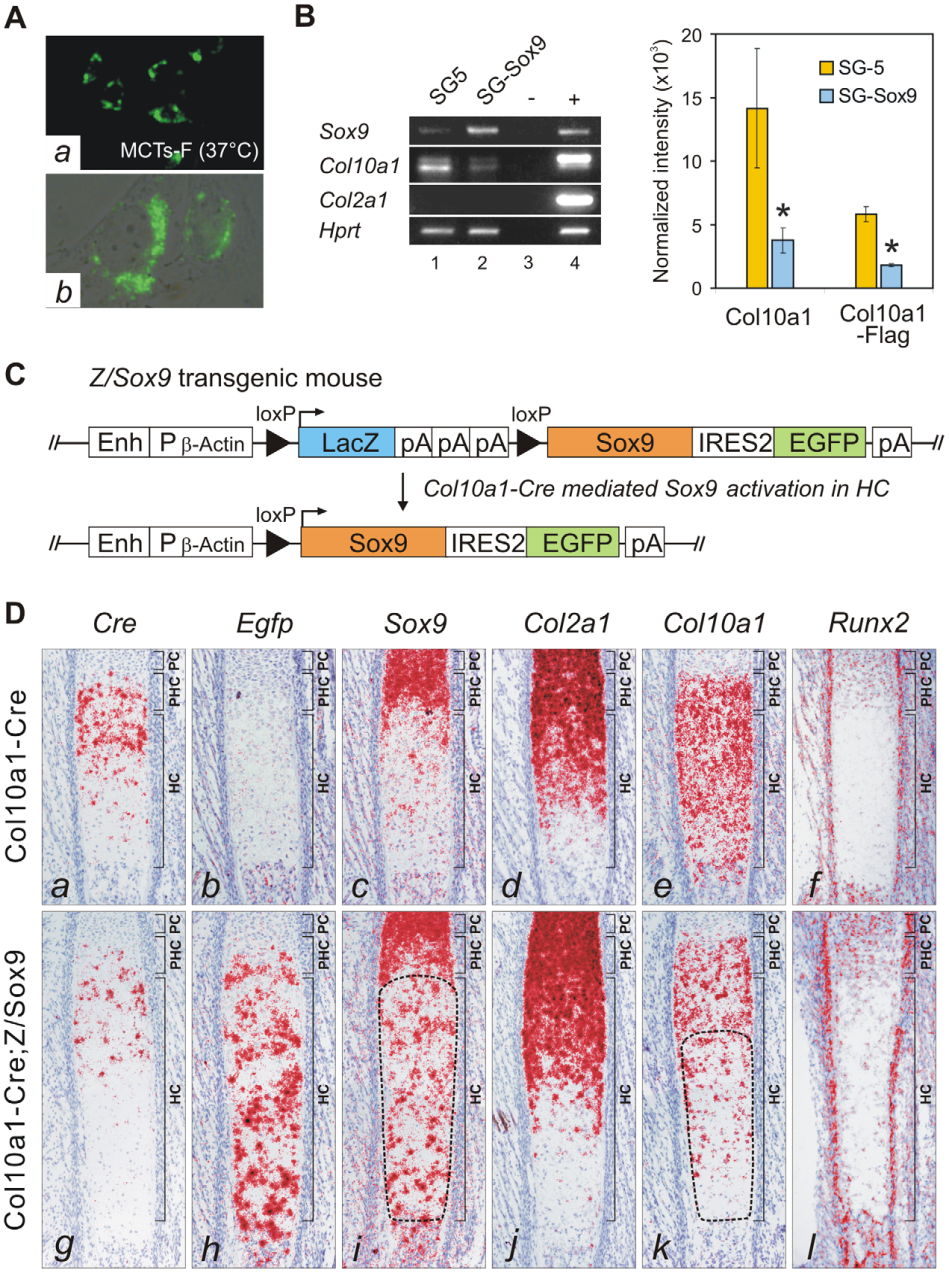
SOX9 directly represses *Col10a1* in hypertrophic chondrocytes. (*A*) Clonal cell line MCTs-F expressing the Col10^Flag^-E was established from MCTs. (*a*) Immunohistochemistry indicated up-regulated Flag-tagged collagen X in growth arrested MCTs-F cells. (*b*) Flag signal in the cytosolic vesicles. (*B*) MCTs-F cells transfected with SOX9 expression vector (pSG-Sox9), or empty vector (pSG5). Expression of *Col10a1* and *Col10a1-Flag* (36 bp longer in amplicon) was significantly reduced upon exogenous *Sox9* expression (*, *p*≤0.02). No RNA (−) or RNA from E16.5 Col10^Flag^-E expressing fetus (+) was used as control. (*C*) Strategy for activating ectopic *Sox9* expression in hypertrophic chondrocytes. The pZ/Sox9 construct consists of a *LacZ* and *Sox9*-*EGFP* expression cassette attached to a chicken *β-Actin* promoter (P_β-Actin_)/CMV enhancer (Enh). The *Sox9*-*EGFP* cassette is expressed after Cre-mediated recombination. (*D*) Cre-activated ectopic *Sox9* expression in the hypertrophic zone of costal cartilage in *Col10a1-Cre*;*Z/Sox9* compound mutants at E17.5. Ectopic *Sox9* expression (*i*, encircled) down-regulated *Col10a1* (*k*, encircled) and *Cre* (*g*) in hypertrophic chondrocytes, especially in the extended hypertrophic zone. The reduction of *Col10a1* is not associated with change in *Runx2* expression (*l*) in hypertrophic chondrocytes. PC, proliferating chondrocytes; PHC, prehypertrophic chondrocytes; HC, hypertrophic chondrocytes.

To test whether SOX9 negatively regulates *Col10a1*, we established a cell line from hypertrophic chondrocytes MCTs which expressed the Col10^Flag^-E transgene at the non-permissive (growth-arrest) temperature (Figure 5*A*). Similar to previous findings for dedifferentiated chondrocytic cells MC615, over-expression of SOX9 in MCTs did not transactivate endogenous *Col2a1* [20]. However, SOX9 over-expression significantly down-regulated expression of both endogenous *Col10a1* and the exogenous *Col10^Flag^-E* reporter (Figure 5*B*) supporting the notion that SOX9 is a negative regulator of *Col10a1*. To study the regulation *in vivo*, SOX9 was ectopically expressed in hypertrophic chondrocytes in mice. Mice expressing *Cre* recombinase inserted into the endogenous *Col10a1* gene (*Col10a1-Cre*) [21] were crossed with transgenic mice carrying a single copy of a Cre-inducible Sox9-IRES-EGFP expression construct (Z/Sox9) [22] (Figure 5*C*). In *Col10a1-Cre*;*Z/Sox9* mice, *Sox9* and the linked *Egfp* reporter gene were activated in the hypertrophic chondrocytes at E17.5 in the anterior ribs (Figure 5*D*, *h*, *i*). These mice displayed an expanded hypertrophic zone and reduced *Col10a1* expression (Figure 5*D*, *e*, *k*). Interestingly, we also found that transcription of *Cre* from the *Col10a1* locus was reduced in *Col10a1-Cre*;*Z/Sox9* mice (Figure 5*D*, *a*, *g*). *Col2a1* expression in *Col10a1-Cre*;*Z/Sox9* mice hypertrophic chondrocytes was not up-regulated suggesting the reduction of *Col10a1* expression in these cells was not because of a reversion to a more immature state (Figure 5*D*, *d*, *j*). In the ribs *Runx2* was expressed predominantly in osteoblasts, the perichondrium flanking the hypertrophic chondrocytes, and in prehypertrophic chondrocytes (Figure 5D, *f*). Expression was low in hypertrophic chondrocytes. There was no significant change in *Runx2* expression in prehypertrophic and hypertrophic chondrocytes in *Col10a1-Cre*;*Z/Sox9* mice (Figure 5D, *l*). Collectively, these data suggest that SOX9 negatively regulates *Col10a1* gene expression independent of *Runx2*.

### GLI factors bind and regulate *Col10a1* in proliferating chondrocytes

**Figure 6:**
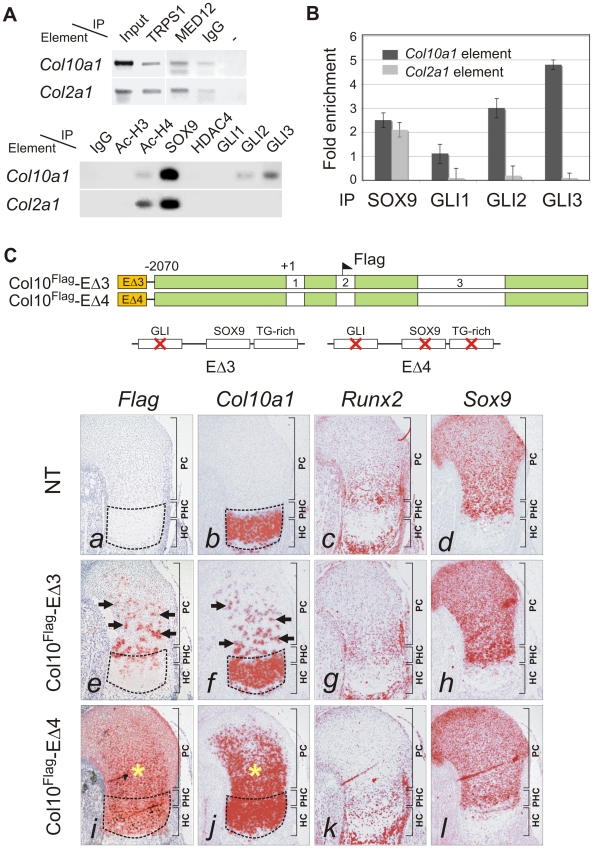
GLI as SOX9 partner in *Col10a1* repression. (*A*) ChIP assays with CCL cells (*upper panel*) and E13.5 mouse limbs (*lower panel*) using various antibodies. The products were amplified with primers for element A of *Col10a1* or enhancer element of *Col2a1* in intron 1. Extract was immunoprecipitated with rabbit IgG or without antibody (−) as negative reference. Extract was directly used in PCR as positive control (input). (*B*) The specific interaction of GLI with *Col10a1* element A was validated in CCL cells by quantitative ChIP assay with real-time PCR. The degree of enrichment was calculated by normalization of readouts to that of IgG control. (*C*) The expression of *Col10a1* mini-genes with mutation in the GLI site was examined by *in-situ* hybridization of proximal humeri in E15.5 fetuses. For transgene with GLI site mutation alone (Col10^Flag^-EΔ3), islands of misexpression (*Flag* and *Col10a1*, arrows) were detected in the non-hypertrophic chondrocytes in most (7/10) expressing fetuses (*e–h*), while the rest showed milder mis-expression (not shown). For the transgene with a mutation in both the GLI site and the SOX9/TG-rich motif (Col10^Flag^-EΔ4), the majority of expressing fetuses (3/5) showed strong and extensive mis-expression (*) (*i–l*), while the rest showed relatively little mis-expression. Non-transgenic littermate (NT) is shown as control (*a–d*). The prehypertrophic and hypertrophic zones are encircled. PC, proliferating chondrocytes; PHC, prehypertrophic chondrocytes; HC, hypertrophic chondrocytes.

The specificity of SOX protein action is known to be achieved through interaction with cell-specific partners [23], [24]. We questioned whether concomitant transactivation of *Col2a1* and repression of *Col10a1* by SOX9 in proliferating chondrocytes could be mediated by different combinations of cofactors. ChIP assays in E13.5 mouse limb chondrocytes or CCL cells revealed similar interactions of TRAP230/MED12, a mediator of SOX9 activity [25], and of TRPS1, a GLI3-interacting repressor [26], [27], with both the *Col10a1* element A and the *Col2a1* enhancer (Figure 6*A*, *upper panel*). On the other hand, the transcriptional co-repressor, histone deacetylase HDAC4 [28] immunoprecipitated neither element. GLI1, GLI2 and GLI3 are effectors of Hh signaling which controls chondrocyte proliferation and maturation [29]. GLI1 is a transactivator expressed in proliferating chondrocytes and perichondrial tissue flanking the prehypertrophic and hypertrophic zones [30] whereas GLI2 and GLI3 can act as repressors and are predominantly expressed in non-hypertrophic chondrocytes and are down-regulated in hypertrophic chondrocytes [29], [31]. Since there is a conserved GLI-binding site near the SOX9/TG-rich motif in the same footprint block P3 (Figure 1*C*), we examined whether GLI1, GLI2 and GLI3 can interact with the element A. Strikingly, while SOX9 bound to both the *Col10a1* element A and *Col2a1* enhancer, GLI2 and GLI3 associated with only *Col10a1* element A (Figure 6*A*, *lower panel*). GLI3 interacted the most with element A, while GLI1 interaction was much less. Quantitative ChIP assays confirmed the preferential interaction (Figure 6*B*). From these results we hypothesized that GLI proteins may repress *Col10a1* expression. To test this *in vivo*, we examined the impact on transgene expression of mutating the GLI-binding site in element A (Col10^Flag^-EΔ3). Consistent with our hypothesis, the majority of fetuses (7 out of 10) expressing Col10^Flag^-EΔ3 showed distinct islands of transgene misexpression in non-hypertrophic chondrocytes (Figure 6*C*, *e*, *f*). Mutating all three sites (GLI, SOX9, TG-rich) in the transgene (Col10^Flag^-EΔ4) did not restrict the expansion of the expression domain to proliferating chondrocytes in all the expressing transgenic fetuses obtained (Figure 6*C*, *i*, *j*). Indeed in the majority of these expressing fetuses (3 out of 5), transgene expression extended throughout the entire cartilage zones. Thus mutation of the GLI site alone had a similar derepressing effect as mutating the SOX9/TG-rich motif and mutating all the motifs did not restrict expression but resulted in more extensive mis-expression. This is consistent with a model whereby SOX9 and GLI act cooperatively to repress *Col10a1* transcription.

**Figure 7:**
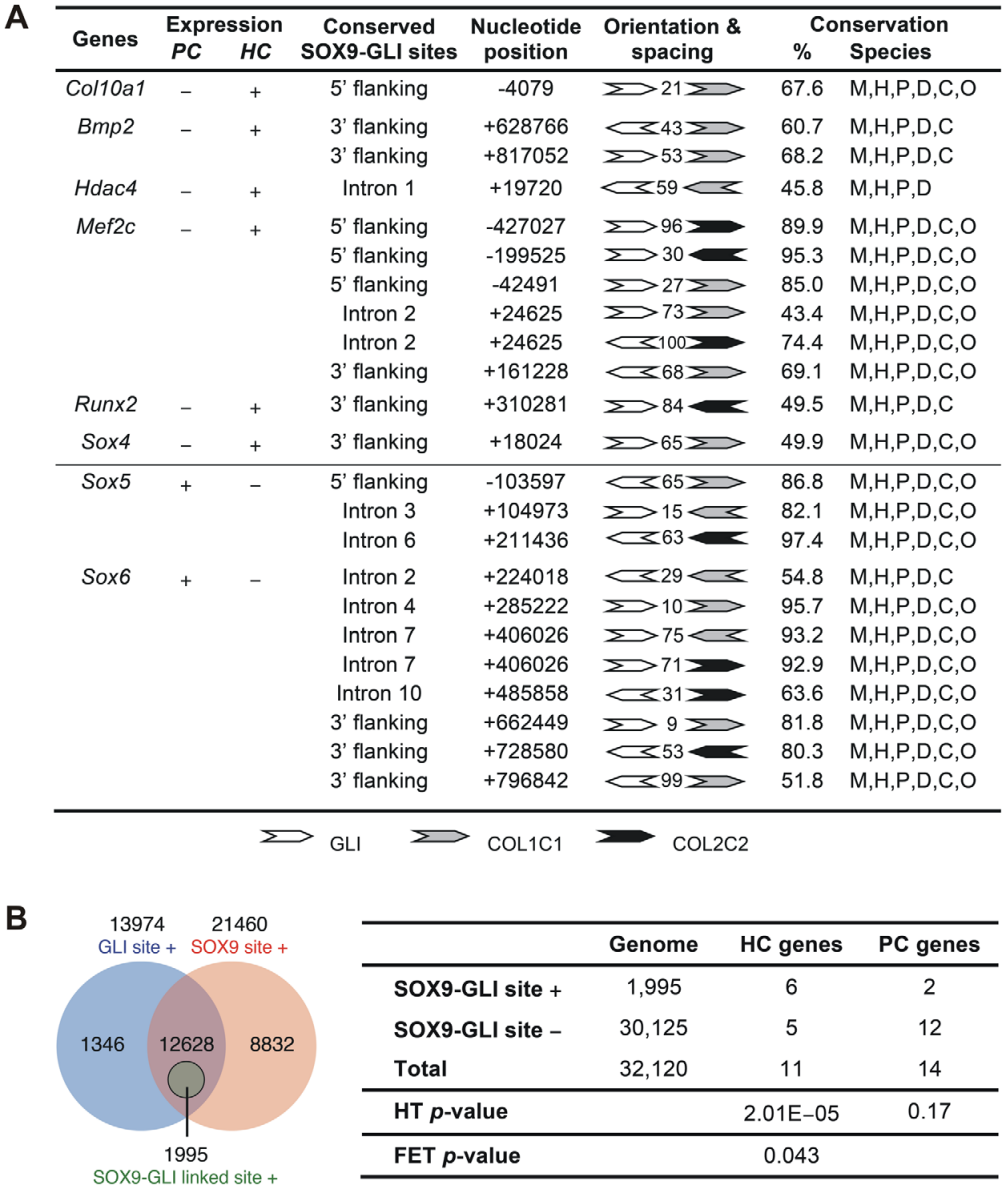
Differential presence of SOX9-GLI sites in proliferating and hypertrophic phase-specific genes. (*A*) Position, orientation, and spacing of the conserved linked SOX9 (COL2C1 and COL2C2) and GLI binding sites within the inter- and intra-genic regions of genes preferentially expressed in proliferating (PC) or hypertrophic (HC) chondrocytes. Numbers between the sites indicate the length of nucleotide spacing. Conservation indicates the percentage of nucleotides and species conserved in the alignments spanning the SOX9 and GLI sites. The alignments were based on multiz30way in UCSC genome browser and were simplified to only include sequences from mouse (M), human (H), chimpanzee (P), canine (D), bovine (C), and opossum (O) genome. Genes tested but found negative include HC genes *Bmp6*, *Cdkn1c*, *Loxl4*, *Spp1*, and *Vegfa* as well as PC genes *Acan*, *Bmp7*, *Col2a1*, *Col9a1*, *Col9a2*, *Col9a3*, *Col11a1*, *Col11a2*, *Fgfr3*, *Fos*, *Mia1*, and *Sox9*. (*B*) Left panel: Venn diagram showing frequency of genes in the genome that contain perfectly conserved SOX9, GLI, or linked SOX9-GLI sites within the inter- and intra-genic regions. SOX9 and GLI sites co-exist but are not linked in the majority of genes. Right panel: Number of HC, PC and all genes in the mouse genome with conserved SOX9-GLI sites and *p*-values from hypergeometric test (HT, for the over-representation of positive HC and PC genes compared to the genome) and one-tailed Fisher's exact test (FET, for the difference in number of positive genes between HC and PC group). The test was based on all genes in the mouse genome (32,120 genes). See also Figure S3 for alignment data of the linked sites.

To assess whether the cooperation of SOX9 and GLI2/3 is a potential common mechanism for restricted or preferential gene expression in hypertrophic chondrocytes, we searched *in silico* for this configuration of binding sites in genes, other than *Col10a1*, that have strong and specific up-regulation in hypertrophic chondrocytes (HC genes) in the growth plate. Six of 11 HC genes analyzed, namely *Col10a1*, *Bmp2*, *Hdac4*, *Mef2c*, *Runx2*, and *Sox4*, possess the linked SOX9 and GLI sites (<100 nt spacing) in the inter- or intragenic conserved non-coding regions (Figure 7*A* and Figure S3). In contrast, these sites were absent from most of the genes tested (12 out of 14) that were expressed in proliferating but not (or down-regulated) in hypertrophic chondrocytes (PC genes). These include known SOX9 targets: *Col2a1*, *Col9a1*, *Col11a2*, *Acan*, and *Mia1* (see Figure 7*A* legend for all negative genes). The exceptions were *Sox5* and *Sox6* (Figure 7*A* and Figure S3). To investigate whether the over-representation of linked SOX9-GLI sites in the HC genes but not the PC genes occurs by chance, we performed a hypergeometric test to calculate the probability of finding 6 or more SOX9-GLI site-containing genes out of 11 genes randomly sampled from the mouse genome. For the HC genes, the results showed that the occurrence of 6 or more genes with associated conserved SOX9-GLI sites is unlikely to occur by chance (*p* = 0.0000201) (Figure 7*B*). For the PC genes, the *p*-value was 0.17, which is comparable to random occurrence. Furthermore the frequency of the presence of SOX9-GLI sites for HC genes (6/11) was significantly higher than that for PC genes (2/14) (Fisher's test *p* = 0.043, one tailed). This suggests that the linked SOX9-GLI sites are preferentially associated with the HC genes.

## Discussion

The positive and negative mechanisms mediating the stage-specific transcription of genes within the growth plate are not well defined, partly because of the difficulty in distinguishing direct effects on transcription from the consequences of abnormal differentiation. In this study we have exploited the specificity of *Col10a1* expression in hypertrophic chondrocytes and the fact that manipulating its expression *in vivo* has no overt effect on differentiation, to dissect these transcriptional controls. We provide new insight into how differentiation stage-specific gene expression is achieved in the growth plate, presenting *in vitro* and *in vivo* evidence that SOX9, in addition to its known role as a transactivator of many genes preferentially expressed in non-hypertrophic chondrocytes, such as *Col2a1*, directly represses expression of *Col10a1* at a stage prior to the onset of hypertrophy and subsequently in proliferating chondrocytes. This discovery extends our understanding of the mechanisms by which SOX9 controls chondrocyte differentiation phase-specific gene expression.

We have identified a conserved regulatory sequence, element A, that acts as an enhancer of *Col10a1* expression in both cultured cells and *in vivo*. This element contains a SOX9 binding sequence that, when bound by SOX9, *represses Col10a1* expression in immature and proliferating chondrocytes. Since *Sox9* is expressed in non-hypertrophic chondrocytes but not in hypertrophic chondrocytes, this repressive action of SOX9 restricts *Col10a1* expression to hypertrophic chondrocytes.

SOX9 has been proposed to direct chondrogenic fate in osteo-chondroprogenitor cells in part by interacting with RUNX2 [32], [33]. SOX9 may inhibit chondrocyte hypertrophy in part via activation of *Bapx1* which represses *Runx2* [34], [35]. Previous *in vitro* and *in vivo* studies suggest that *Col10a1* expression is regulated positively by *Mef2c*, *Runx2/Cbfa1*, and AP-1 members, which are expressed in hypertrophic chondrocytes [19], [36]–[38]. RUNX2 has been shown to directly regulate the expression of *Col10a1* [37]. The element A that we identified contains no conserved consensus RUNX site. The RUNX2 site revealed by Zheng *et al.* [37] is located within a poorly conserved region outside the element. Our data showed that the ectopic expression of *Col10a1* transgene in non-hypertrophic chondrocytes does not require co-expression of *Runx2*. In addition, RUNX2 is not expressed in the costal hypertrophic chondrocytes and cultured hypertrophic chondrocytes MCTs (which is derived from costal cartilage), where *Col10a1* expression is strong. Although real-time PCR showed levels of *Col10a1* was markedly reduced in P1 *Runx2*-null mice [37], hypertrophic chondrocytes with strong *Col10a1* expression do develop in many cartilages in *Runx2* null fetuses [39], [40]. Collectively existing data suggest that RUNX2 together with other factors regulate *Col10a1 in vivo* via promoting chondrocyte hypertrophy or otherwise functions to initiate a cascade of regulatory pathways that sustain *Col10a1* expression in hypertrophic chondrocytes.

Previous *in vitro* studies in chicken have suggested that a combined action of positive and negative DNA elements may contribute to the hypertrophic chondrocyte-specific expression of *Col10a1* [41], [42]; however, these chick *Col10a1* elements are not conserved in mammals. The enhancer element we identified is highly conserved in mammals, but not in chicken, which agrees with previous data [43]. This suggests that in both mammals and chicken, *Col10a1* transcription is restricted to hypertrophic chondrocytes by repression, though by different *cis*-acting elements. In the chicken, this repression may extend to non-chondrogenic cell types [41]. We found no evidence to support such a mechanism in the mouse since when we abolished the interaction of SOX9 with the repressive element, we observed no ectopic *Col10a1* expression in non-chondrogenic cells.

Consistent with a role for SOX9 in repressing *Col10a1 in vivo*, we have shown in *Col10a1-Cre;Z-So*x9 mice, that activation of *Sox9* expression in hypertrophic chondrocytes in costal cartilage caused down-regulation of *Col10a1*. It is also notable that in a recent report where *Sox9* was over-expressed in hypertrophic chondrocytes in transgenic mice, expression of *Col10a1* and the *BAC-Col10-Sox9* transgene appeared reduced in the hypertrophic chondrocytes [44]. In the same report *Sox9* knockdown in cultured chondrocytes did not affect *Col10a1* expression [44]. By contrast Yamashita *et al.* showed that shRNA knockdown of *Sox9* can up-regulate *Col10a1* expression in primary costal chondrocyte culture, and that over-expression of *Sox9* can down-regulate it [35]. These contradictory results may be related to the incomplete elimination of SOX9 protein and the known dosage dependent requirement for SOX9 action. A role for SOX9 as transcriptional repressor of *Col10a1* in non-hypertrophic chondrocytes is consistent with the observation that *COL10A1* expression was up-regulated in cartilage isolated from *SOX9* haploinsufficient campomelic dysplasia patients [33].

**Figure 8:**
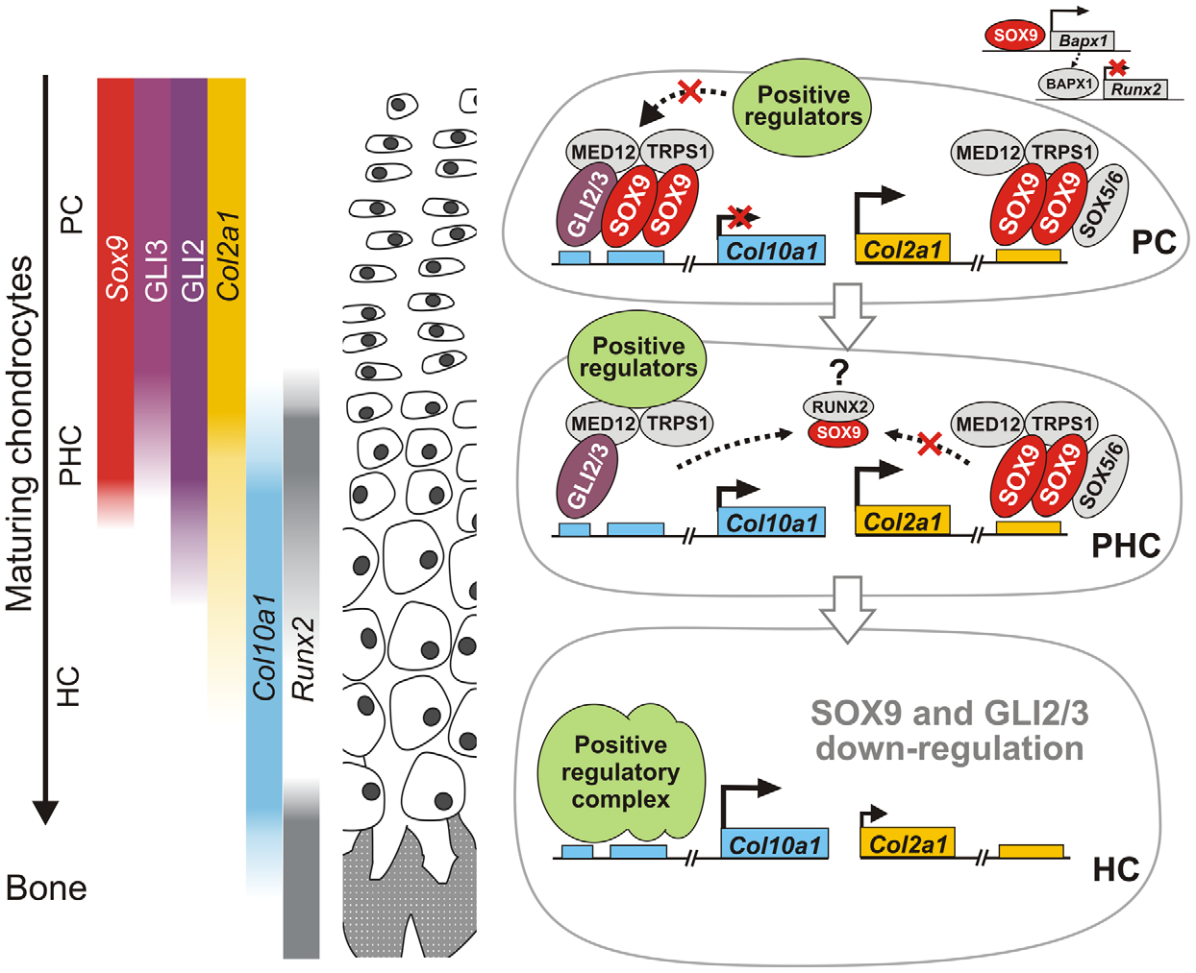
A model for SOX9 control of differentiation phase–specific gene expression in growth plate chondrocytes. As chondrocytes mature during endochondral bone formation, the concomitant transactivation and repression of transcription of differentiation stage-specific genes by SOX9 is exemplified in *Col10a1* and *Col2a1*. *Sox9*, its transactivation target *Col2a1*, as well as GLI2 and GLI3 are preferentially expressed by non-hypertrophic chondrocytes. GLI3 is down-regulated when chondrocytes become prehypertrophic, while *Sox9* and GLI2 expression is down-regulated when chondrocytes reach hypertrophy [8], [9], [29]. Regulation of both *Col10a1* and *Col2a1* involves dimeric binding of SOX9. The GLI factors, and possibly others, depending on the spatial context of their binding sites, are involved in the differential transcriptional control. MED12 and TRPS1 [25]–[27] act as common mediators of transcriptional control. In proliferating chondrocytes, *Col10a1* is repressed through binding of SOX9 and GLI2/3. SOX9 negatively regulates *Runx2* expression via *Bapx1* in proliferating chondrocytes [34], [35]. When chondrocytes mature and become prehypertrophic, RUNX2 binding to SOX9 may reduce the amount available for *Col10a1* [33] and, as GLI3 levels reduce, *Col10a1* is partially released from repression. *Col2a1* transcription may be maintained due to stabilization of SOX9 binding by SOX5/6 [15]. In hypertrophic chondrocytes, as SOX9-associated machinery diminishes, *Col10a1* is completely de-repressed and is highly transactivated by factors such as RUNX2, MEF2C, AP-1. Concomitantly *Col2a1* expression is markedly decreased. SOX9 therefore controls differentiation-phase specific gene expression in chondrocytes through direct and indirect mechanisms. PC, proliferating chondrocytes; PHC, prehypertrophic chondrocytes; HC, hypertrophic chondrocytes.

How may SOX9 act both as a transactivator and a repressor in non-hypertrophic chondrocytes? It has been proposed that interactions between specific partner factors stabilize SOX protein binding to DNA and hence regulate target selection [24], thereby determining cell specification, as exemplified by SOX2 in embryonic stem cells and other systems [45]. Such selective cooperation of protein binding partners may mediate the concomitant positive and negative regulation of SOX9 target genes in the same cell that contributes to specification of the differentiation state (Figure 8). As illustrated in the schematic (Figure 8), we propose that SOX9 mediates repression of *Col10a1* in proliferating chondrocytes by selective cooperation with GLI factors. Together with its role in activating *Col2a1* and other matrix genes, SOX9 therefore plays an important role in maintaining chondrocytes in an immature non-hypertrophic state. RUNX2 by contrast promotes chondrocyte hypertrophy.

SOX9 cannot regulate chondrocyte differentiation appropriately without sonic hedgehog (Shh), which mediates the generation of chondrogenic precursor cells [46], and Indian hedgehog (Ihh), which regulates their proliferation and maturation [47]. GLI proteins are the effectors of Hh signaling. Double knockout mutants indicate that GLI2 has overlapping functions with GLI1 and GLI3 in skeletal and CNS development [48], [49]. Binding of Hh to its receptor, Patched, blocks the proteolytic processing of the GLI transcription factors from active (GLI^A^) to repressive (GLI^R^) forms, and the balance between these forms modulates hedgehog target gene expression [50]. In the growth plate, GLI2^A^ can positively regulate chondrocyte hypertrophy and control vascularization of the hypertrophic cartilage in endochondral ossification [29], [51]. GLI3, which acts mainly as a repressor, has been suggested to inhibit chondrocyte hypertrophy [29], [52] and it is interesting that the highest interaction of element A was with GLI3. Mau *et al.* reported that *Gli2/3* null mutations altered the expression domain of collagen X, but it was not possible to distinguish whether this was due to a direct effect on *Col10a1* transcription or more general perturbation of hypertrophy [29]. How the GLI factors interact with other regulatory factors or genes in the chondrocyte differentiation program is not clear. Our results are consistent with cooperation between SOX9 and the Hh signaling pathway and suggest that SOX9 acts in synergy with GLI2 and GLI3, probably their repressive forms GLI^R^, to repress transcription in chondrocytes. Thus, reduced synergy between GLI^R^ and SOX9 may explain the accelerated chondrocyte hypertrophy seen when *Ptch1* is inactivated [53]. Over-representation of the SOX9-GLI paired consensus in a number of genes that are preferentially expressed in hypertrophic chondrocytes and not in proliferating chondrocytes, suggests that SOX9 may use this partnership to repress transcription of several genes in other chondrocyte types. However this partnership may not be the exclusive mechanism by which SOX9 acts to repress expression in chondrocytes.

Hattori *et al.* have recently shown that SOX9 directly represses *Vegfa* in cultured primary chondrocytes [44] by interacting with the 5′ untranslated region of the gene. This agrees with our findings of a repressive role of SOX9, however, we found no linked SOX9-GLI binding sites near the *Vegfa* gene and a recent SOX9 ChIP-on-chip study reported no *in vivo* interaction in *Vegfa* exon 1 [54].

A different mode by which SOX9 may repress gene expression in chondrocytes has been proposed by Huang *et al* [55]. In their model, SOX9 negatively regulates *Ccn2* expression in non-hypertrophic chondrocytes via binding to overlapping binding sites for SOX and TCF/LEF, thereby interfering with binding of a TCF/LEF/β-catenin transactivation complex. Reduction of SOX9 upon hypertrophy allows this TCF/LEF/β-catenin complex to activate *Ccn2* expression [55]. However, *Ccn2* is also expressed in resting zone chondrocytes in the epiphyses of the growth plate and it is not clear why SOX9 does not repress the gene in these cells. We identified a conserved TCF consensus site in *Col10a1* element A, but this is unlikely to interfere with SOX9 binding since it is located 59 bp downstream of the functional SOX9-GLI motif, unlike in *Ccn2* where the SOX and TCF/LEF sites overlap (Figure 1*C*). This suggests that the model proposed by Huang *et al.* does not apply to *Col10a1* element A-mediated repression of transcription.

It is also possible that SOX9 and GLI cooperate to activate or repress transcription depending on context. While our data implicate a cooperation of SOX9 with GLI factors in transcriptional repression, this association between SOX9 and GLI may not be restricted to negative regulation. Amano *et al.* have recently reported that GLI2 cooperates with SOX9 to transactivate the *Pthlh* gene (also known as *PTHrP*) in chondrocyte culture without direct binding to the gene [56]. However the expression patterns of *Sox9* and *Pthlh* are mutually exclusive in the developing growth plate, *Pthlh* being expressed mainly in the perichondrium and only at extremely low levels in proliferating chondrocytes [57], [58]. This contradiction may reflect differences between *in vitro* assays and regulation *in vivo*, and it is also unclear whether the expressed GLI2 was processed to a repressor form or not in these cells. The observed stimulation of *Pthlh* promoter activity in the cultured chondrocytes could therefore be attributable to over-expression of GLI2 which persisted largely as the activated form GLI2^A^. However this report does raise the possibility of a context dependent SOX9-GLI partnership that mediates either transactivation or repression.

*Sox9* has been suggested to act upstream of *Sox5* and *Sox6* in chondrogenesis [6], [46]. The presence of conserved SOX9–GLI sites in the *Sox5* and *Sox6* genes suggests their expression in proliferating chondrocytes may be positively controlled via cooperation of SOX9 with GLI^A^ or GLI1, an activator that reinforces GLI^A^ function. Hence, the roles played by SOX9 in transcriptional regulation may be determined by context—partnering with GLI^A^/GLI1 favours transactivation, with GLI^R^ favours repression. Alternatively, as discussed above, the mode of regulation might depend on whether intermediate factors are present to interfere with the SOX9-GLI interaction. Interestingly, while there is a linked SOX9-GLI motif in *Sox6*, a conserved TCF site occurs between the SOX9 and GLI sites (Figure S3). Cooperation between SOX9 and TCF/LEF/β-catenin might therefore abrogate cooperative repression by SOX9 and GLI and transactivate *Sox6* in proliferating chondrocytes.

Validation of these different modes of cooperative regulation by SOX9 and GLI factors *in vivo* would require the generation and analyses of compound null or conditional knockout mutants; however, the consequent dysregulation of chondrogenesis and impact on cell survival would make it impossible to distinguish changes in transcriptional control from effects on differentiation. For example, *Sox9* is essential for chondrogenesis and *Sox9* conditional null chondrocytes undergo apoptosis and as a consequence, hypertrophy with the characteristic activation of *Col10a1* expression, fails to occur [4], [7], [59]. Because inactivation of *Col10a1* does not disrupt the chondrogenic program, it provides an ideal system and tools to interrogate the transcriptional controls governing specificity of gene expression within the growth plate, independent of changes in chondrocyte differentiation. Important questions to be addressed in future are the identities and diversity of SOX9 partnerships and how the activity of SOX9 and its partners is modulated. In early myogenic differentiation, SOX9 and Smad3 have been reported to prevent premature expression of α-sarcoglycan gene expression by synergistic repression in a transforming growth factor β dependent manner [60] suggesting negative transcriptional regulation by SOX9 and its partners may be more common. The transactivation of *Matn1* in chondrocytes by SOX9 can be modulated by combined action of L-SOX5, SOX6 and NFI factors [61]. Whether control of transcription by the SOX9-GLI partnership can be modulated by additional factors is an important question to be addressed in future.

In summary, our study implicates a complex regulatory function for SOX9 whereby it acts with different partners to orchestrate activation and repression of transcription in the chondrogenic differentiation pathway. Mutations in human *SOX9* cause the skeletal malformation syndrome campomelic dysplasia which is attributed to the disruption of the chondrogenic differentiation program because of failure to express SOX9 target genes. This interpretation may need to be revised to include inappropriate expression of genes normally repressed by SOX9.

## Materials and Methods

### Bioinformatics analysis

*COL10A1* sequences were aligned using LAGAN and PipMaker. The conserved SOX9 binding sites (COL2C1 and COL2C2) [10] were identified by rVISTA. The UCSC Mouse 30-Way Multiz Alignment data and a custom Perl script were used to identify human-mouse perfectly conserved SOX9, GLI, and TCF sites with a maximum spacing of 100 bp in the inter- and intra-genic non-coding regions. Conservation percentage of sequence spanning the SOX9 and GLI sites was calculated based on the number of perfectly matched nucleotides among all the aligned species (mouse, human, chimpanzee, canine, bovine, and opossum). From the 32,120 genes in the mouse genome, the number of genes containing conserved linked SOX9-GLI sites was found and used as the reference frequency of such genes in the genome. Whether the frequency of the presence of conserved SOX9-GLI sites in HC or PC genes exceeded this reference frequency was assessed by the hypergeometric distribution. The difference in the frequency of the presence of SOX9-GLI sites in the HC and PC genes was assessed by the Fisher's exact test.

### Cell culture and transfection

Hypertrophic chondrocyte cell line MCTs (gift of Véronique Lefebvre [62]) was transfected with pCol10^Flag^-E, pSG-Sox9 (gift of Peter Koopman), or pSG5 expression vector using Fugene 6 (Roche). Expression of the exogenous collagen X from pCol10^Flag^-E in MCTs cells was examined by immunohistochemistry using anti-Flag M2 antibody (Sigma). CCL (gift of James Kimura [63]), MCT3T3-E1, and COS-1 cells were cultured in DMEM (Invitrogen) containing 10% FCS (Wisent) at 37°C at 5% CO_2_. MCTs cells were normally cultured at 32°C at 5% CO_2_ for expansion. Prior to assays, MCTs cells were cultured at 37°C for 1 day to induce growth arrest [62].

### DNase I footprinting

A 300 bp DNA fragment within element A, corresponding to −4240 to −3935 bp of the mouse *Col10a1*, was used as probe. [γ-^32^P]ATP-labeled probes were incubated with nuclear extracts from CCL, MCTs, MCT3T3-E1, or COS-1 cells in the presence of poly(dA⋅dT) at room temperature, followed by DNase I digestion and denaturing PAGE.

### Electromobility shift assays

COS-1 nuclear extracts over-expressing SOX9 and RUNX2 were pre-incubated with poly(dI⋅dC) or poly(dG⋅dC) at room temperature, followed by reaction with [γ-^32^P]ATP-labeled probes with or without the presence of nucleotide competitors, or antibodies for SOX9 (gift of Peter Koopman [64]) and OSF2/RUNX2 (gift of Gerard Karsenty [65]), then subjected to non-denaturing PAGE at room temperature. The sequences of oligonucleotide COL2C1 and OSE2 were as previously described [10], [65].

### Col10^Flag^ transgenic mice

The mouse *Col10a1* element A, a 640 bp-fragment located between −4.2 and −3.6 kb, was cloned at the 5′ end of the pCol10^Flag^, ^previously known as FColX^ [^18^] consisting of −2070 to +7176 bp mouse *Col10a1* genomic sequence^,^ to generate pCol10^Flag^-E. The SOX9, TG-rich motif, and GLI binding sites in pCol10^Flag^-E were mutated to generate the single site mutants – respectively pCol10^Flag^-EΔ1, pCol10^Flag^-EΔ2, and pCol10^Flag^-EΔ3. All of these 3 motifs in pCol10^Flag^-E were mutated to generate pCol10^Flag^-EΔ4.

### *Col10a1-Cre*;*Z/Sox9* compound mutants

A 4.8 kb fragment of mouse genomic DNA (from 82 bp upstream of the start of transcription of *Sox9* to 1119 bp downstream of the polyadenylation sequences), including the *Sox9* coding region, its two introns and 1.1 kb of 3′ flanking DNA, together with an IRES2-EGFP (Clontech) sequence inserted between the *Sox9* stop codon and the polyadenylation site (at +3237 bp), was cloned downstream of the loxP-flanked βgeo/3xpA of the pCall2 vector (gift of Andras Nagy [66]) to create the pZ/Sox9 expression vector. pZ/Sox9 was transfected into 129/SvEv-derived L4 embryonic stem (ES) cells by electroporation and ES clones containing a single copy of the transgene were injected into blastocysts followed by crossing of chimeras with C57BL/6N mice to generate the mouse line *Z/Sox9*. A mouse line carrying a single copy of pZ/Sox9 was generated which was then crossed with *Col10a1-Cre* mice [21] to obtain compound mutants.

### Chromatin immunoprecipitation

CCL cell lysates or 13.5 dpc mouse limb tissue lysates were cross-linked followed by lysis and sonication to yield 200–500 bp DNA fragments then immunoprecipitation with antibodies against acetylated histone H3/H4 (Ac-H3, Ac-H4) (Upstate), HDAC4 (Abcam), GLI1, GLI2, GLI3 (all from Santa Cruz), SOX9 (gift of Robin Lovell-Badge [67]), TRAP230/MED12 (gift of Robert Roeder [25]), or TRPS1 (gift of Yasuteru Muragaki [26]). The target elements in *Col10a1*, *Col2a1*, or *Crygb* genes were amplified by real-time or standard PCR.

### Gene expression analysis

*In-situ* hybridization was performed as previously described [9]. The probes used were pRK26 for *Col10a1* [68], pWF21 for the *Flag* sequence [18], p88 for full-length *Sox9* (gift of Peter Koopman), pBS-Cbfa1-Sh for full-length *Runx2* (gift of Gerard Karsenty), pWF98 for full-length *Egfp*, pBSII-Cre-frag for *Cre* (gift of Andrew Groves), and pNJ61 for *Col2a1* [9]. Gene expression in cell culture was analyzed by RT-PCR.

For additional details of all experiments, see the Text S1.

## Supporting Information

Figure S1 Additional gene/protein expression data and EMSA. (*A*) Expression of *Col2a1* and *Col10a1* in CCL, MCTs, and MC3T3-E1 was analyzed by RT-PCR. Controls were no RNA (−) or RNA from mouse E17.5 embryo (+). Expression of *Col10a1* was found only in MCTs. (*B*) Western blot analysis showed that both SOX9 and RUNX2 were highly expressed in CCL but weakly in MCTs. (*C*) Expression of SOX9 in nuclear (lane 3) and cytoplasmic (lane 4) fractions of pcDNA-Sox9 transfected COS-1 cells was compared with CCL (lane 1) and untransfected COS-1 (lane 2) nuclear extracts by Western blotting. (*D*) Expression of RUNX2 in nuclear (lane 3) and cytoplasmic (lane 4) fractions of pcDNA-Cbfa1 transfected COS-1 cells was verified by Western blotting. (*E*) A diagram showing the sequences of oligonucleotides which contained wild-type, mutant SOX9 and/or TG-rich motif for EMSA. Only the mutated nucleotides were shown in the sequence of competitors. (*F*) Intact DNA-binding property of SOX9 in expressing nuclear extract was tested by its interaction with the *COL2A1* enhancer probe (COL2C1) in EMSA, in which the retarded band was challenged with SOX9 antibody (lane 4), unlabeled COL2C1 (lane 5–6), or *Col10a1* element A C10C1 oligonucleotides (lane 7–8). The triangles represent increasing concentration of competitors from 10× (lane 5,7) to 100× (lane 6,8) excess.
(TIF)

Figure S2 *In vivo* transcriptional activity of *Col10a1* element A. (A) Expression pattern of Col10^Flag^-E in E15.5 transgenic fetus (mid-sagittal plane) examined by *in-situ* hybridization using digoxigenin-labeled probes. *Flag* expression was detected in the hypertrophic chondrocytes of cervical pedicle (*a*) and developing vertebrae (*b*). Weak expression was detected in the trabecular bone of palate (*c*). No expression was found in the nucleus pulposus (np) of the intervertebral disc (*b*), immature costal cartilage (*g*, cc), brain (*h*), hair papillae (*i*), lung (*m*), aorta (*n*), or myocardium (*o*). Expression of *Col10a1* is shown for comparison (*d–f*, *j–l*, *p–r*). No expression was found in erythrocytes or nucleated blood cells (* in *n* and *q*). (B) In Col10^Flag^ transgenic fetus, strong expression of *Flag* was found in the ossifying zone of pedicle (*a*, circled) and the trabecular bone of palate (*b*, circled). Weak signal was detected in the prehypertrophic zone of the pedicle cartilage (*a*, arrow). No expression was identified in tissue other than cartilage and bone. Expression of *Col10a1* is shown for comparison (*c–d*).
(TIF)

Figure S3 Alignment data from SOX9-GLI site analysis. Multispecies alignment of regions spanning the linked SOX9-GLI sites are shown along with chromosomal location (Chr), identical nucleotides (.), insertion or deletion (-), and unaligned positions ( = ).
(PDF)

Text S1 Additional details of experiments.
(DOC)
