# Supplementary figures and images for: SOX9 Governs Differentiation Stage-Specific Gene Expression in Growth Plate Chondrocytes via Direct Concomitant Transactivation and Repression
Source: PLoS Genet. 2011 Nov 3;7(11):e1002356. doi: 10.1371/journal.pgen.1002356 (PMC3207907; doi:10.1371/journal.pgen.1002356)

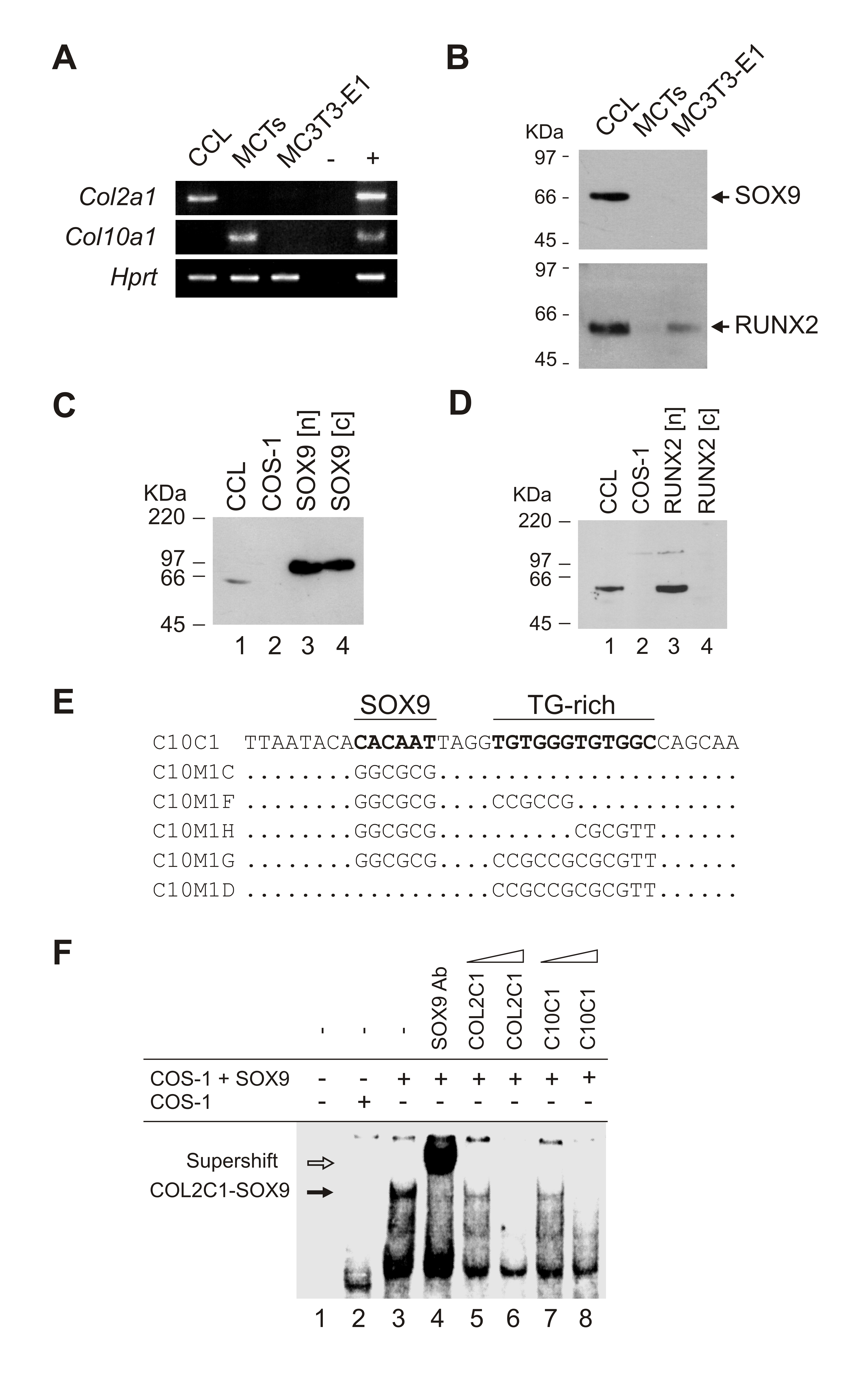

Supplement: Figure S1 — Additional gene/protein expression data and EMSA. (A) Expression of Col2a1 and Col10a1 in CCL, MCTs, and MC3T3-E1 was analyzed by RT-PCR. Controls were no RNA (−) or RNA from mouse E17.5 embryo (+). Expression of Col10a1 was found only in MCTs. (B) Western blot analysis showed that both SOX9 and RUNX2 were highly expressed in CCL but weakly in MCTs. (C) Expression of SOX9 in nuclear (lane 3) and cytoplasmic (lane 4) fractions of pcDNA-Sox9 transfected COS-1 cells was compared with CCL (lane 1) and untransfected COS-1 (lane 2) nuclear extracts by Western blotting. (D) Expression of RUNX2 in nuclear (lane 3) and cytoplasmic (lane 4) fractions of pcDNA-Cbfa1 transfected COS-1 cells was verified by Western blotting. (E) A diagram showing the sequences of oligonucleotides which contained wild-type, mutant SOX9 and/or TG-rich motif for EMSA. Only the mutated nucleotides were shown in the sequence of competitors. (F) Intact DNA-binding property of SOX9 in expressing nuclear extract was tested by its interaction with the COL2A1 enhancer probe (COL2C1) in EMSA, in which the retarded band was challenged with SOX9 antibody (lane 4), unlabeled COL2C1 (lane 5–6), or Col10a1 element A C10C1 oligonucleotides (lane 7–8). The triangles represent increasing concentration of competitors from 10× (lane 5,7) to 100× (lane 6,8) excess. (TIF) [file pgen.1002356.s001.tif]

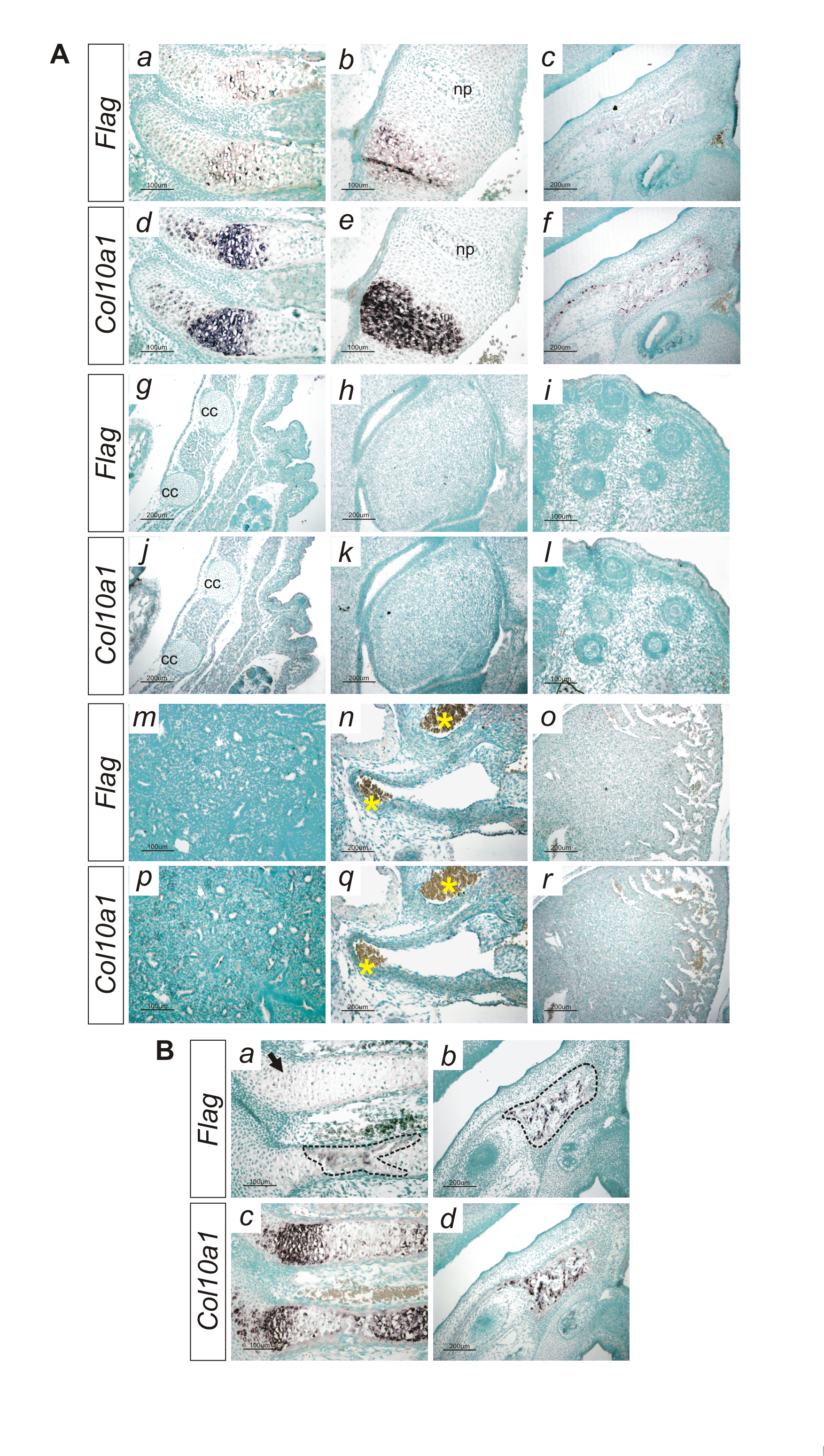

Supplement: Figure S2 — In vivo transcriptional activity of Col10a1 element A. (A) Expression pattern of Col10Flag-E in E15.5 transgenic fetus (mid-sagittal plane) examined by in-situ hybridization using digoxigenin-labeled probes. Flag expression was detected in the hypertrophic chondrocytes of cervical pedicle (a) and developing vertebrae (b). Weak expression was detected in the trabecular bone of palate (c). No expression was found in the nucleus pulposus (np) of the intervertebral disc (b), immature costal cartilage (g, cc), brain (h), hair papillae (i), lung (m), aorta (n), or myocardium (o). Expression of Col10a1 is shown for comparison (d–f, j–l, p–r). No expression was found in erythrocytes or nucleated blood cells (* in n and q). (B) In Col10Flag transgenic fetus, strong expression of Flag was found in the ossifying zone of pedicle (a, circled) and the trabecular bone of palate (b, circled). Weak signal was detected in the prehypertrophic zone of the pedicle cartilage (a, arrow). No expression was identified in tissue other than cartilage and bone. Expression of Col10a1 is shown for comparison (c–d). (TIF) [file pgen.1002356.s002.tif]
