# Supplementary material for: SOX9 Governs Differentiation Stage-Specific Gene Expression in Growth Plate Chondrocytes via Direct Concomitant Transactivation and Repression
Source: PLoS Genet. 2011 Nov 3;7(11):e1002356. doi: 10.1371/journal.pgen.1002356 (PMC3207907; doi:10.1371/journal.pgen.1002356)
Supplement: Figure S3 — Alignment data from SOX9-GLI site analysis. Multispecies alignment of regions spanning the linked SOX9-GLI sites are shown along with chromosomal location (Chr), identical nucleotides (.), insertion or deletion (-), and unaligned positions ( = ). (PDF) [file pgen.1002356.s003.pdf]

```
>chr10:34105562-34105595 (Col10a1) [34bp]

            GLI                         SOX9
            >>>>>>>                     >>>>>>
Mouse       ACACCCAGAATAAAAATAGTTTAATACACACAAT
Human       .........G............T.A.T.......
Chimpanzee  .........G............T.A.T.......
Cow         ..T..T................T.A.T.......
Dog         ..T...C...............T.A.T.......
Opossum     .A.T-.................T.A.T.T.....

>chr2:134007701-134007756 (Bmp2) [56bp]

            GLI                                               SOX9
            <<<<<<<                                           >>>>>>
Mouse       TGGGTGTTCCTGTCACTGTCACCCTAAAATCCAGGGCAGGCTGGAAGTTGCACAAT
Human       .........------......T.T.G.C..T....C...T.CT.............
Chimpanzee  .........------......T.T.G.C..T....C...T.CT..........G..
Cow         .........------..........G.C..TG..CC..ATTCT.........T...
Dog         .........------..........G.C...A...C...C.CTA............

>chr2:134195987-134196052 (Bmp2) [66bp]

            SOX9                                                         GLI
            <<<<<<                                                       <<<<<<<
Mouse       ATTGTGTCCCACTTCCTCGGAGTGATATCATCATTACACAT--CTCACTGATAAAAATAGATGGGTGG
Human       .......AA..T.....AAA........T.......T.T.ACC...........T.............
Chimpanzee  .......AA..T.....AAA........T.......T.T.ACC...........T.............
Cow         ........A..T....GAAA........T.....C.T.T.AAC....T......T.GCCTT.......
Dog         .C......A..T.....AAA........G.....CG..T.ACC...........T.GC..........

>chr1:94025250-94025321 (Hdac4) [72bp]

            SOX9                                                               GLI
            >>>>>>                                                             >>>>>>>
Mouse       CACAATAAATAATAGACACACAATGAGCCAAGAGACCCACATGCAAGTTGGAAA--AGATCAGAATAACACCCA
Human       .........a..-----G..TG....A.A....A.TTA.T..A..T.....T----...CAGA..C........
Chimpanzee  -........a..-----G..TG....A.A....A.TTA.T.CA..T.....T.GAC...CAGA..C........
Dog         g....c..ca..G.A--G..TTG.....A....AGTTGGGC.ATCT.....T..AC...CT.C.G.G.T.....

>chr13:83216167-83216275 (Mef2c) [109bp]

            GLI                                                                                                    SOX9
            >>>>>>>                                                                                                >>>>>>
Mouse       CCACCCATTAGCCTCAACACTACACACTGATGCTATACTTACACCTCACTCGTTATGTATTTACTTTTTATATAACTAAGCAGCACCCTGTCATAAATGAAATATTCAT
Human       .....................--...T..C.................G.............................................................
Chimpanzee  .....................--...T..C.................G.............................................................
Cow         .....................--...T..C.................G.............................................................
Dog         .....................--...T..C.................G.............................................................
Opossum     ...A........T...GT...--C..T..C..........................A....................................................


>chr13:83443669-83443711 (Mef2c) [43bp]

            SOX9                                GLI
            >>>>>>                              <<<<<<<
Mouse       ATTCATTTGACCATGACCAATAAATAATTTATGAACTGGGTGT
Human       .............CA............................
Chimpanzee  .............CA............................
Cow         .............CA............................
Dog         .............CA............................
Opossum     .............CA............................


>chr13:83600703-83600742 (Mef2c) [40bp]

            GLI                               SOX9
            >>>>>>>                           >>>>>>
Mouse       CCACCCATACCTAGGACTGGTGCCACAGCCAGACCACAAT
Human       ................GC......................
Chimpanzee  ................GC......................
Cow         ......G.........GC......................
Dog         ................GC......................
Opossum     ................GCA......TG.............


>chr13:83667713-83667825 (Mef2c) [113bp]

            SOX9                                                                                                               GLI
            <<<<<<                                                                                                             >>>>>>>
Mouse       ATGAATGTTACTTAAACATCATTTTTATGAA---AATATAATACGTGGAGACTTTGTT----AGAGAATAGTTTTGTTTTACCT-GTAGCATTTTTAAAACTCAAACTTT-CTAAACACCCA
Human       ..........TA....G......C..TAC..CAG.....G..GTT.T....G...A..TCAG...AT.A.......G.....A.AA..A......G....TCTT.....C-T..C.......
Chimpanzee  ..........TA....G......C..TAC..CAG.....G..GTT.T....G...A..TCAG...AT.A.......G.....A.AA..A......G....TCTT.....C-T..C.......
Cow         ......A..TTAC...GG.....C..TAC..CAA...GCG..GTT.TC...G...A..TCAG...A-.A.......G.....A.AA..A......G.....C.CT.....-T..TG..T...
Dog         ........G.TG..C.G..T...C..TAC..CAA...---..GTTAT....G...A..TCAT...ATCA.......G....TA.AA..A..C...G.....C.T.....C-T..C...T..-
Opossum     .CA.G....GGAAG.TT....CAC..TAC..TAG..C....AGTTCT.GC.G..G...CA---..AT.A.A..A..G.....A.AA..A......G...TA..C.....CTT..GCT.T...


>chr13:83667819-83667904 (Mef2c) [86bp]

            GLI                                                                             SOX9
            >>>>>>>                                                                         >>>>>>
Mouse       ACACCCAACACCACAGTAATTTCTTGCTTCTTTTAAAATTGAAGTCCTTTTTTACCCTACTGATGCCTCCTAGTGTCTCCCACAAT
Human       .........TTT..........T.........C........................A..................T..A......
Chimpanzee  .........TTT..........T.........C........................A..................T..A......
Cow         G..T.....T.T.....................A.......................A..................T..A......
Dog         ...T..-..T.T.....................A.......................A..............C...T..A......
Opossum     CT.T...GTT.T.TGA.C.......A......C..........C.............A..................T..A......


>chr13:83804422-83804502 (Mef2c) [81bp]

            GLI                                                                        SOX9
            <<<<<<<                                                                    >>>>>>
Mouse       TGGGTGGCAGAAGCCAAAAGACCTTATTAACGAGGCTATATTTTTCTTAACTTGATCTGCAATCCAGAATTAGACCACAAT
Human       .................G.............T...........................A......C..............
Chimpanzee  .................G.............T...........................A......C..............
Cow         .........A.......G........C................................A......C........TG....
Dog         .........A.......G........C....C.....G.....................A......C............TC
Opossum     .................G...TT...C.C..TG.A..C..-.....C......T.....A...TTCC......TT.T.---


>chr17:44641295-44641391 (Runx2) [97bp]

            GLI                                                                                           SOX9
            >>>>>>>                                                                                       <<<<<<
Mouse       ACACCCATAGGTACAGAGACTCCATGTGGCTAAATGGAATAGCTTTTGGTGGGAA-AT--ACAAGGTGTTTGTACAACCTGTATATTCTTTGCCATGAAT
Human       ........TT.G.AG.....ATA-....A...T..AT....T....AT...C-TT-T.TG.AGG.A.....A......T..C.....A....G.......
Chimpanzee  ........TT.G.AG.....ATA-....A...T..AT....T....AT...C-TT-T.TG.AGG.A.....A......T..C..........G.......
Cow         ...T....TT.G.AG.....A---....A...T..AT...GT....AT...ATTTGGGGG.AG..C......................C...G....CCC
Dog         GTC.....TT.G.GG.....A.A-....A..GC..AT....C....AT...CTTT-T.TG.AGG.CC....AC..G...A........C.C.TGC.....


>chr13:29027527-29027604 (Sox4) [78bp]

            SOX9                                                                      GLI
            <<<<<<                                                                    <<<<<<<
Mouse       ATTGTGACGGTCAAATAGGCCCCTCTGTGGGGTGT-AT--TCTGCCTTGGATGCAGCATTCTGACAGGCTCTTCTGGGTGT
Human       .......A..C.................A......C.C--.......CA.....T..T......T..A.AT..........
Chimpanzee  .......A..C.................A......C.C--.......CA.....T..T......T..A.AT..........
Cow         ..CCAA.T........G..................CCC--.......CA....GT..T...C..T..A......A.A....
Dog         ....CA.T..C...G....................C.C--.......CA.....T..T......T..AA............
Opossum     G-..CACTA.C.......CT..T.G.AAA..ACC.C..CAC...A..CA....T...T......TT.C...C.T......C
```

```
>chr6:143946564-143946639 (Sox5) [76bp]

            GLI                                                                   SOX9
            <<<<<<<                                                               <<<<<<
Mouse       TGGGTGGTACCCTTTACAAATCACCTTAATGGTGGCTCGGTGACAGCGAAACAACCTTCAAGATCGCCTCATGAAT
Human       ....................C........CT.......A.......T..................A...T......
Chimpanzee  ....................C........CT.......A.......T..................A...T......
Cow         ....................C........CT.......A.......T..................A...T......
Dog         ....................C........CT.......A.......T..................A...T......
Opossum     ....................C........CT..A....A.......TA............G....A...T......


>chr6:144053027-144053054 (Sox5) [28bp]

            GLI                   SOX9
            >>>>>>>               <<<<<<
Mouse       ACACCCACACGGAAGAGTCTGCATTGTG
Human       ..........T...C.............
Chimpanzee  ..........T...C.............
Cow         ..........TT..C.............
Dog         ..........T...C.............
Opossum     .....T....T...C...T.........


>chr6:144261597-144261674 (Sox5) [78bp]

             SOX9                                                                   GLI
             <<<<<<                                                                 >>>>>>>
Mouse        ATTGTGACTTCAGACAGTGCCTTCAAGGGTCTCTACTGTACCGAATTGAGGTCACTGCAAGACAATGCCCAACACCCA
Human        ....................T...............................................T.........
Chimpanzee   ....................T...............................................T.........
Cow          ....................T...............................................T.........
Dog          ....................T.........................................................
Opossum      ....................T.........................................................


>chr7:122950543-122950584 (Sox6) [42bp]

            SOX9                                GLI
            >>>>>>                              >>>>>>>
Mouse       CACAATGCCCAGGAACCCTC-ACAAAGGATTCCAGTCCACCCA
Human       ......A..........TCT-........CA............
Chimpanzee  ......A..........TCT-........CA............
Cow         ......A.....A.....CT-.....A...G............
Dog         ......A.TT........CT-.........G............
Opossum     ..A..-------------=========================


>chr7:122889339-122889361 (Sox6) [23bp]

             GLI              SOX9
             >>>>>>>          >>>>>>
Mouse        ACACCCAGGCACTGCTGCACAAT
Human        .......................
Chimpanzee   .......................
Cow          .......................
Dog          .......................
Opossum      ............G..........


>chr7:122768535-122768622 (Sox6) [88bp]

             GLI                                                                            SOX9
             >>>>>>>                                                                        >>>>>>
                                                                                                SOX9
                                                                                                <<<<<<
Mouse        ACACCCAATGACGGGCAAGAATAATGATGAAAAAACAAGAGATCTATGTCTATGAAACCGTGTTGGACAAAAT-GGGCCATTCATTGTG
Human        .........................T..............A................................-...............
Chimpanzee   .........................T..............A................................-...............
Cow          .........................T..............A................................-...............
Dog          .........................T..............A................................-...............
Opossum      .......G.................T..............AT...............TA..............G...............


>chr7:122768535-122768618 (Sox6) [84bp]

             GLI                                                                            SOX9
             >>>>>>>                                                                        >>>>>>
Mouse        ACACCCAATGACGGGCAAGAATAATGATGAAAAAACAAGAGATCTATGTCTATGAAACCGTGTTGGACAAAAT-GGGCCATTCAT
Human        .........................T..............A................................-...........
Chimpanzee   .........................T..............A................................-...........
Cow          .........................T..............A................................-...........
Dog          .........................T..............A................................-...........
Opossum      .......G.................T..............AT...............TA..............G...........


>chr7:122688703-122688746 (Sox6) [44bp]

            GLI                                    SOX9
            <<<<<<<                                >>>>>>
Mouse       TGGGTGTGGGTGCTC-TTGTGGTTTATATTCTGGTCTCTATTCAT
Human       ..........CA...T.CA....C.....................
Chimpanzee  ..........CA...T.CA...CC...........T.........
Cow         ....C.....CA...T.CA....C.....................
Dog         .........ACA...C.CA....C.....................
Opossum     ........T.CA...T.CA....C..CG......C....CCC...


>chr7:122512112-122512133 (Sox6) [22bp]

            SOX9           GLI
            <<<<<<         <<<<<<<
Mouse       ATTGTGTGTGCTGAGTGGGTGT
Human       ......................
Chimpanzee  ......................
Cow         ........CT............
Dog         ......................
Opossum     .....T......T.........


>chr7:122445981-122446046 (Sox6) [66bp]

            SOX9                                                       GLI
            >>>>>>                                                     >>>>>>>
Mouse       ATTCATGAAAGGCGGCTTGCTTGCATAAATAAATTAAAAGATGCAAAACTAAAGTTTAAACACCCA
Human       ..................................................................
Chimpanzee  ..................................................................
Cow         ............A...........................C.........................
Dog         ........................................CA........................
Opossum     .....G.....C.A.......CA...C.....................AG..G.C...........


>chr7:122377719-122377830 (Sox6) [112bp]

            GLI                                       TCF                                                           SOX9
            <<<<<<<                                   >>>>>>>                                                       >>>>>>
Mouse       TGGGTGGAAGGCCAGACAATGGAGCGCTATATTTGGTTTGACAACAAAGGCCTCAAGAAAGATTTGATAGTATGGGGATGGCCGGGTAGGCGGTACAAGAATCCTGCACAAT
Human       .....................T..T..............C..........G.C.T................GCA.......T...A....A.CG..G..TG-.A.......
Chimpanzee  .....................T..T..............C..........G.C.T................GCA.......T...A....A.CG..G..TG-.A.......
Cow         .....................T............................G.C.T................GC.............A.......G.G...TG-.A.......
Dog         .......G.............T............................G.C.C......GC........GG.....A...G...AG.....-G.GG..TG-.A.......
Opossum     ..T...C.......C.....ATCT.A.......C...GCA..G.....A.G.C.T.A.G.......T...G.A...A......A.AACACACA.CATGCCTG-.CAT.....
```
