# Supplementary material for: SOX9 Governs Differentiation Stage-Specific Gene Expression in Growth Plate Chondrocytes via Direct Concomitant Transactivation and Repression
Source: PLoS Genet. 2011 Nov 3;7(11):e1002356. doi: 10.1371/journal.pgen.1002356 (PMC3207907; doi:10.1371/journal.pgen.1002356)
Supplement: Text S1 — Additional details of experiments. (DOC) [file pgen.1002356.s004.doc]

**Text S1**

**Bioinformatics analysis.** Genomic sequences flanking 100 kb both 5’ and 3’ of the transcription start site in *COL10A1* were extracted from human ([*Homo sapiens*](http://www.ensembl.org/Homo_sapiens/)NCBI 36), chimp (*Pan troglodytes* PanTro 2.1), bovine (*Bos taurus* Btau 2.0), canine (*Canis familiaris* CanFam 2.0), mouse ([*Mus musculus*](http://www.ensembl.org/Mus_musculus/)NCBI m36), and opossum (*Monodelphis domestica* MonDom 4.0) at Ensembl (http://www.ensembl.org). LAGAN and rVISTA are provided online at http://www-gsd.lbl.gov/vista [1] and PipMaker is provided at http://bio.cse.psu.edu/pipmaker [2]. Identification and sequence conservation of the SOX9-GLI binding site was assessed by 30-Way Multiz Alignment of the Mouse July 2007 Assembly (mm9) available in the UCSC Genome Bioinformatics web site (http://genome.ucsc.edu). COL2C1 site – 5’CACAAT-3’; COL2C2 site – 5’ATTCAT-3’; GLI site – 5’A[CA]ACCCA-3’.

**Cell culture and transfection.** Cell lines used in this study: COS-1 - monkey fibroblasts; CCL - rat chondrosarcoma; MCTs - immortalized mouse rib chondrocytes; MC3T3-E1 - mouse pre-osteoblasts. MCTs cell transfection was performed at 32 °C at a DNA concentration of 1ug/ml medium using Fugene 6 (Roche) under manufacturer’s protocol. To stably express an exogenous *Col10a1* reporter, MCTs cells were co-transfected with the linearized pCol10^Flag^-E and pPGK-Hygro^R^ in a 10:1 w/w ratio and cultured in the presence of hygromycin-B (300 ug/ml, Roche). Independent hygromycin-B resistant clones were characterized for transgene expression using RT-PCR for *Col10a1*. The Col10^Flag^-E expressing clones were then transiently transfected with *Sox9* (pSG-Sox9, gift of Peter Koopman), or empty (pSG5) expression vector for 24 h. RT-PCR was performed on RNA prepared from the transfected cells after culturing for 24 h at 37 °C.

**DNase I footprinting.** The nuclear extracts were prepared according to Scott *et al*. [3]. 2 x 10^7^ cells were resuspended in 400 ul cold buffer A for 15 min at 4 °C and disrupted by addition of 25 ul 10% NP40 with vigorous vortexing for 10 s. The nuclear pallet was resuspended in 50 ul buffer C and rocked for 15 min. After centrifugation for 5 min, the nuclear extract was collected as a supernatant. DNase I footprinting assay was performed as described by Leblanc *et al*. [4]. Briefly, 10 ng probe, which was labeled on one strand by [γ-^32^P]ATP (GE Healthcare), was incubated with 100 ug nuclear extract in the presence of 1 ug poly(dA⋅dT) (GE Healthcare) and 60mM KCl (BDH) for 30 min at room temperature. After the incubation, the samples were subject to DNase digestion at 0.0025 to 0.4 Kunitz U DNase I (Invitrogen) for 1 min. Guanine/adenine (G/A) ladders were generated by partial chemical cleavage of the labeled probe using piperidine formate (Sigma). The DNase-digested samples and G/A ladders were heat-denatured and fractionated in 6% denaturing polyacrylamide gel. Signal was captured by Storm830 phosphoimager (Molecular Dynamics). The probe was generated by PCR using primers 5’CGTAATGGCAGCTAATTG-3’ and 5’CCTTACTTCAGCATACAG-3’ with mouse *Col10a1* element A as template.

**Electromobility shift assays (EMSA).** SOX9 and RUNX2 proteins were expressed in COS-1 cells by transient expression of pcDNA-Sox9 (gift of Peter Koopman) and pcDNA-Cbfa1 vectors, respectively, and the nuclear extracts were prepared as described above. EMSA were performed as described by Scott *et al.* [3] with modifications. In brief, 10 ug of nuclear extract was mixed with 1ug of poly(dI⋅dC) (GE Healthcare, for SOX9-expressing extract) or poly(dG⋅dC) (GE Healthcare, for RUNX2-expressing extract). The mixture was pre-incubated at room temperature for 10 min and then 1ng [γ–^32^P]ATP-labeled probe was added followed by incubation for 15 min. Antibody was added to the incubation reaction to identify the transcription factor present in the complexes. To verify sequence-specific interactions, 10-100x unlabeled double-stranded oligonucleotides was added into the pre-incubation mixture. The reactions were then fractionated by 4% non-denaturing polyacrylamide gel electrophoresis at room temperature. The signal was captured by Storm830 phosphoimager (Molecular Dynamics). The sequences of oligonucleotide COL2C1 and OSE2 were the same as previously described [5,6].

**Transgenic mice expressing Col10^Flag^ mini-genes.** The SOX9 binding site in pCol10^Flag^-EΔ1 was mutated from 5’CACAAT-3’ to 5’GGCGCG-3’ using a PCR-based method. The TG-rich motif in pCol10^Flag^-EΔ2 was similarly mutated from 5’TGTGGGTGTGGC-3’ to 5’CCGCCGCGCGTT-3’. The GLI binding site in pCol10^Flag^-EΔ3 and pCol10^Flag^-EΔ4 was mutated from 5’AACACCCAGA-3’ to 5’CCAGGTAGTC-3’. Transgenic mice harboring the various *Col10a1* constructs were generated by pronuclear injection of one-cell zygotes of F1 (C57BL/6N x CBA/Ca) hybrid mice as previously described [7]. Expressing E15.5 fetuses were identified by RT-PCR for Flag sequence using total RNA from the hind limbs.

**Genotyping of transgenic mice.** The *Z/Sox9* and *Col10a1-Cre* mice, as well as the compound mutants, were genotyped by PCR using the following primers:

*Cre*  5’GGACATGTTCAGGGATCGCCAGGCG-3’

5’GCATAACCAGTGAAACAGCATTGCTG-3’

*Egfp*  5’GTTCTTCTGCTTGTCGGCCA-3’

5’ATGGTGAGCAAGGGCGAGGA-3’

**Chromatin immunoprecipitation (ChIP).** ChIP assays (Upstate Biotechnology ChIP kit) were performed according to the manufacturer’s protocol with some modifications. Briefly, 1% formaldehyde was added to CCL or 10T1/2 cell lysates, or tissue lysates extracted from developing mouse limbs at E13.5 for 10 min, followed by quenching with 125 mM glycine. After PBS washing, cells were lysed and sonicated in 1 % SDS, 10 mM EDTA, 50 mM Tris, pH 8.0 and protease inhibitor cocktail (Roche) to yield 200–500 bp DNA fragments. Lysates were immunoprecipitated with antibodies. Control serum, rabbit IgG, or no antibody was used as controls. Immunoprecipitates were washed and incubated at 65 °C for over 6 h to reverse the formaldehyde crosslink. After treatment with proteinase K (Roche), DNA was purified and amplified by standard PCR for 25 cycles with *Taq* DNA polymerase (Invitrogen) using the following primer pairs:

*Col10a1* 5’GGCCTCCTGTTTCACGTAGAAT-3’

5’CTGTTTACCGTTTGCTTTGTTAGTC-3’

*Col2a1* 5’ TTCCAGATGGGCTGAAACC-3’

5’ATTGTGGGAGAGGGGGGTC-3’

*Crygb* 5’TTTTCTCATTGATGGCAACAACA-3’ 5’GCTGAATGGTTTACTCTCATTCTTTCT-3’

Real-time PCR analysis was carried out using SYBR green and fold enrichment along with standard errors were calculated using the following formulas:

ΔCt_(target)_ = ΔCt_(input)_–ΔCt_(target)_

ΔCt_(neg control)_ = ΔCt_(input)_–ΔCt_(neg control)_

ΔSD_(target)_ = √(SD^2^_(input)_+SD^2^_(target)_)/n

ΔSD_(neg control)_ = √(SD^2^_(input)_+SD^2^_(target)_)/n

ΔΔCt = ΔCt_(target)_ –ΔCt_(neg control)_

ΔΔSD = √(ΔSD^2^_(target)_+ΔSD^2^_(neg control)_)

Fold Change = 2^(ΔΔCt)^

Fold Change_(error)_ = log2 (ΔΔSD * Fold Change)

**RT-PCR.** Total RNA was isolated from the cells using the guanidium isothiocyanate extraction. To generate the cDNA, 1 ug of total RNA was reverse transcribed using 500 ng oligo-dT, and 200 U SuperScript II in 1x first strand buffer (Invitrogen) for 1 hour at 42 °C. PCR was then performed using 1/20 volume of the cDNA with 5U of recombinant *Taq* DNA polymerase (Invitrogen) and 100 ng of the following gene-specific primers:

*Sox9* 5’AACGCGGAGCTCAGCAAG-3’

5’TCTCGCTTCAGATCAACT-3’

*Sox18* 5’TGCCGACGAGTTGCGCAT-3’

5’TGACGCTGCAGCGAAGAC-3’

*Col2a1* 5’AGATCTCGAGGGTGTCAGGGCCAGGATGCCC-3’

5’GCCCTTGCAGGACT CCCATC-3’

*Col10a1* 5’GCTTTCCTGGCTTCCTCTGC-3’

5’CGCATACCCTA CTTCATAAC-3’

*Hprt* 5’CCTGCTGGATTACATTAAAGCACTG-3’

5’GTCAA GGGCCATATCCAACAACAAC-3’

All PCR was performed at 30 cycles at an annealing temperature of 54 °C. One fourth of the PCR product was analyzed by 2 % TAE agarose gel electrophoresis. Band intensity was measured by calculating the peak area in the lane profile plot generated from Scion Image, release beta 4.0.3. The mean and the standard deviation were calculated from triplicates of experiments.

**Immunohistochemistry.** Cells were fixed with 4 % paraformaldehyde in PBS for 30 min, treated with methanol for 15 s, and then rinsed 3 times in PBS for 5 min each. After blocking with 5 % goat serum for 30 min, the cells were incubated with mouse monoclonal anti-Flag M2 antibody (Sigma) in 1:50 dilution at 4 °C overnight. Cells were washed twice with PBS for 5 min each and incubated in goat anti-mouse FITC-conjugated antibody (DAKO) in 1:50 dilution for 30 min. Cells were rinsed 3 times with PBS for 5 min each and the slides were mounted in Vectorshield mounting medium (Vector Laboratories).

**References**

1. Loots GG, Ovcharenko I, Pachter L, Dubchak I, Rubin EM (2002) rVista for comparative sequence-based discovery of functional transcription factor binding sites. Genome Res 12: 832-839.

2. Schwartz S, Zhang Z, Frazer KA, Smit A, Riemer C, et al. (2000) PipMaker--a web server for aligning two genomic DNA sequences. Genome Res 10: 577-586.

3. Scott V, Clark AR, Docherty K, Harwood AJ (1994) The Gel Retardation Assay. Methods in Molecular Biology, vol31: DNA-prNA-protein Interactions: Principle and Protocols: Humana Press Inc., Totowa, NJ.

4. Leblanc B, Moss T, Kneale GG (1994) DNase I Footprinting. Methods in Molecular Biology, vol30: DNA-prNA-protein Interactions: Principle and Protocols: Humana Press Inc., Totowa, NJ.

5. Bell DM, Leung KK, Wheatley SC, Ng LJ, Zhou S, et al. (1997) SOX9 directly regulates the type-II collagen gene. Nat Genet 16: 174-178.

6. Ducy P, Zhang R, Geoffroy V, Ridall AL, Karsenty G (1997) Osf2/Cbfa1: a transcriptional activator of osteoblast differentiation. Cell 89: 747-754.

7. Leung KK, Ng LJ, Ho KK, Tam PP, Cheah KS (1998) Different cis-regulatory DNA elements mediate developmental stage- and tissue-specific expression of the human COL2A1 gene in transgenic mice. J Cell Biol 141: 1291-1300.
